# Angiogenesis-independent VEGF signaling enhances exercise capacity by increasing fat oxidation in mice fed sulfur amino acid-restricted diets

**DOI:** 10.1016/j.isci.2025.114148

**Published:** 2025-11-20

**Authors:** Charlotte G. Mann, Michael R. MacArthur, Jing Zhang, Songlin Gong, Jenna E. AbuSalim, Craig J. Hunter, Wenyun Lu, Thomas Agius, Alban Longchamp, Florent Allagnat, Joshua D. Rabinowitz, James R. Mitchell, Katrien De Bock, Sarah J. Mitchell

**Affiliations:** 1 Department of Health Sciences and Technology, ETH Zurich, 8092 Zurich, Switzerland; 2 Department of Chemistry, Princeton University, Princeton, NJ 08544, USA; 3 Lewis-Sigler Institute of Integrative Genomics, Princeton University, Princeton, NJ 08544, USA; 4 Ludwig Princeton Branch, Princeton University, Princeton, NJ 08544, USA; 5 Department of Molecular Biology, Princeton University, Princeton, NJ 08544, USA; 6 Department of Vascular Surgery, Lausanne University Hospital (CHUV), 1005 Lausanne, Switzerland; 7 Transplant Center, Department of Surgery, Massachusetts General Hospital, Harvard Medical School, Boston, MA 02114, USA; 8 Center for Engineering in Medicine, Department of Surgery, Massachusetts General Hospital, Harvard Medical School, Boston, MA 02114, USA; 9 Department of Molecular Metabolism, Harvard T.H. Chan School of Public Health, Boston, MA 02115, USA

**Keywords:** metabolomics, metabolic flux analysis, diet

## Abstract

Dietary restriction of the sulfur-containing amino acids methionine and cysteine (SAAR) has numerous metabolic benefits including enhanced body composition and insulin sensitivity. Many of these benefits parallel those associated with endurance exercise. How SAAR impacts skeletal muscle remains largely unexplored. Here, we demonstrate that one week of SAAR in sedentary young male mice increases endurance exercise capacity. SAAR increased lipid oxidation at rest, delaying the onset of carbohydrate utilization during exercise. SAAR increased expression of fatty acid catabolism genes, especially in glycolytic muscle, leading to increased fatty acid circulatory turnover flux and muscle β-oxidation. Reducing lipid uptake from circulation through endothelial-cell-specific CD36 deletion attenuated the running phenotype. Inhibition of VEGF signaling prevented improved exercise performance following SAAR, independent of angiogenesis. These results support a role for angiogenesis-independent VEGF signaling and endothelial cell CD36-dependent fatty acid transport in the regulation of endurance exercise capacity by mediating muscle substrate availability.

## Highlights

• Short-term sulfur amino acid restriction increases exercise capacity in male mice
• SAAR prolongs systemic fatty acid utilization during exercise
• Glycolytic muscle metabolism boosts fat oxidation for ATP production
• VEGF inhibition blunts exercise phenotype after SAAR

## Graphical abstract

**Figure:**
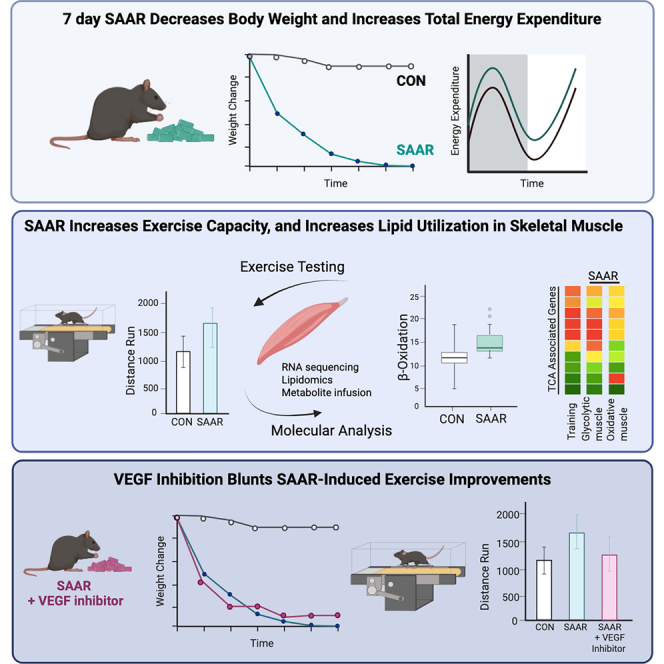

## Introduction

Methionine restriction (MR) was first reported in the early 1990s to extend lifespan of rodents when consumed over the duration of life.^1^^,^^2^ Also known as sulfur amino acid restriction (SAAR), this dietary intervention restricts the sulfur-containing amino acids methionine and cysteine. Unlike calorie restriction, the gold standard intervention for increasing lifespan and health span in various model organisms,^3^^,^^4^^,^^5^ SAAR does not require any restriction in food intake, and the diet is consumed *ad libitum.*^1^^,^^2^ SAAR elicits strong metabolic responses and has been shown to improve body composition and reverse insulin resistance.^1^^,^^2^ Already, after fed only weeks rather than months, SAAR increases thermogenesis via uncoupled respiration in brown adipose tissue (BAT), while also increasing lipolysis and oxidative phosphorylation in white adipose tissue (WAT) and liver.^6^^,^^7^^,^^8^ Interestingly, the effects of this short-term SAAR in skeletal muscle have been less well studied. Given skeletal muscle’s essential role in whole-body energy homeostasis and physical performance, understanding how SAAR influences muscle function is critical. Emerging evidence suggests that SAAR may induce molecular changes in muscle that are consistent with enhanced oxidative capacity.^9^^,^^10^ Among these, angiogenesis has been identified as a potential adaptive response.^11^

Previous research has shown that SAAR increases angiogenesis in skeletal muscle^11^; however, it remains unclear whether these changes translate into functional improvements in exercise performance. Notably, it has been reported that muscle vascularization correlates with mitochondrial density and oxidative phosphorylation capacity^12^ and endurance exercise stimulates angiogenesis in skeletal muscle.^13^ Angiogenesis is a crucial adaptive response in both developmental and pathophysiological conditions characterized by insufficient oxygen and nutrient supply.^14^ Endurance exercise is one of the few non-pathological settings of vascular expansion during adulthood. During angiogenesis, transcription factors like activating transcription factor 4 (ATF4) are induced by various stimuli including mechano-stress responses or the integrated stress response (ISR). Activators of the ISR include endoplasmic reticulum (ER) stress and amino acid deprivation.^15^^,^^16^ These adaptive responses play key roles in regulating muscle metabolism and vascular density by enhancing vascular endothelial growth factor (VEGF) levels.^13^^,^^17^^,^^18^^,^^19^^,^^20^ VEGF-A is the primary regulator of angiogenesis. By binding to the receptor VEGFR2, VEGF-A initiates a cascade of signal transduction involving mediators that facilitate endothelial cell (EC) migration, proliferation, and vessel formation.^21^ VEGF signaling also induces changes in energy metabolism, promoting increased glucose uptake and glycolysis in ECs to meet the energy demands of migration.^22^ The role of the other VEGF isoforms is less understood. Initially considered to passively regulate angiogenesis by scavenging VEGFR1,^23^ research suggests that VEGF-B may actively modulate EC fatty acid uptake.^24^^,^^25^^,^^26^^,^^27^^,^^28^^,^^29^

Given SAAR’s known effects on energy metabolism, we hypothesized that SAAR could induce skeletal muscle adaptations similar to those seen with endurance exercise, including mitochondrial remodeling, improved substrate utilization, and potentially vascular remodeling. This study builds upon our prior work in which longer-term (2–4 weeks) SAAR induced skeletal muscle angiogenesis.^11^ Here, we aimed to define the earliest time point at which metabolic reprogramming and functional improvements begin to emerge. To address this gap mechanistically, we focused on CD36, a fatty acid transporter critical for lipid metabolism, and FGF21, a key endocrine regulator of energy balance, both of which are responsive to nutrient stress. Endothelial CD36, in particular, facilitates transendothelial lipid transport and may play a crucial role in matching substrate delivery to the elevated energetic demands of muscle during SAAR-enhanced endurance.^30^^,^^31^^,^^32^To test this hypothesis, we subjected mice to one week of a SAAR diet then measured their exercise endurance capacity and molecular and physiological responses to the diet.

Here, we demonstrate that this short-term SAAR induces exercise-like adaptations in glycolytic muscle, improving endurance exercise capacity in young sedentary male mice. These effects potentially depend on VEGF signaling, revealing a novel link between dietary amino acid restriction and exercise performance.

## Results

### Short-term SAAR induces shifts in metabolism and increases endurance exercise capacity in young sedentary male mice

**Figure 1:**
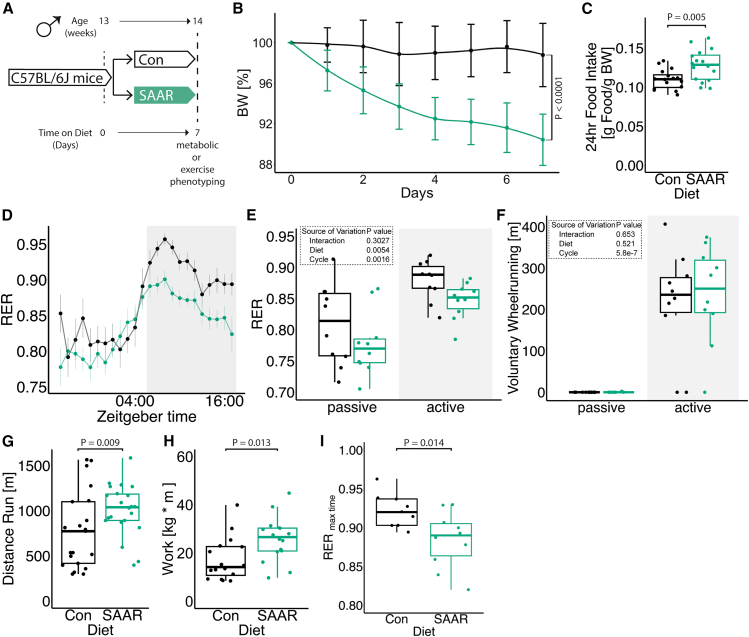
Short-term SAAR induces shifts in metabolism and increases endurance exercise capacity in young sedentary male mice (A) Experimental set up and color scheme used throughout Figures 1 and S1. (B) Body weight trajectory over time, shown as percent of starting body weight (*n* = 10) of male mice given *ad libitum* access to sulfur amino acid-restricted (SAAR) versus control (Con) diet for seven days. (C) Food intake expressed as grams of food per gram of body weight per mouse within a 24 h period (*n* = 16) of male mice given *ad libitum* access to SAAR versus Con diet on day seven. (D and E) (D) Sable systems indirect calorimetry measurements of respiratory exchange ratios (CO_2_ emission/O_2_ consumption, VCO_2_/VO_2_, RER) over a 24 h period (*n* = 10) and (E) the quantified average RER during a 12 h–12 h light-dark cycle (*n* = 10/group) of male mice given *ad libitum* access to SAAR versus Con diet on day seven. (F) The average wheel running in meter during a 12 h–12 h light-dark cycle (*n* = 10) of male mice given *ad libitum* access to SAAR versus Con diet on day seven. (G) Distance ran in meter during a one-time maximal endurance test (*n* = 20) of male mice given *ad libitum* access to SAAR versus Con diet on day seven. (H) Work performed during a one-time maximal endurance test (*n* = 20) of male mice given *ad libitum* access to SAAR versus Con diet on day seven, depicted as kg bodyweight times meters ran. (I) Quantification of RER at maximal exercise time, during a one-time maximal endurance test, performed on a metabolic treadmill (Harvard Apparatus) (*n* = 16), of male mice given *ad libitum* access to SAAR versus Con diet on day seven. (B–F) represent data from mice that were not subjected to endurance running, (G and H) represent data from mice subjected to maximal endurance testing. All data are shown as mean, and error bars indicate SD unless otherwise noted; *p* values indicate the significance of the difference by Student t test or two-way ANOVA with Sidak multiple comparisons test between diets or diet and cycle (indirect calorimetry); significance is determined by *p* < 0.05. Each dot represents an individual mouse. See also Figure S1 and Table S2.

To evaluate the effect of short-term SAAR on systemic metabolism, young sedentary male mice were fed a SAAR diet for seven days (experimental scheme, Figure 1A). Seven days of SAAR reduced body weight by an average of 8.65% (Figures 1B, S1A, and S1B) and increased food intake (Figures 1C and S1C), consistent with previous reports on SAAR.^1^^,^^2^ Lean to fat mass ratio was not changed by SAAR (Figure S1D).

To investigate systemic metabolic changes following seven days of SAAR, we performed indirect calorimetry using metabolic cages with voluntary running wheels. After seven days, mice on SAAR had elevated energy expenditure (EE) during both the active and passive phases (Figures S1E and S1F). We did not normalize EE to body weight or lean mass, as this can introduce statistical artifacts when the independent variable (body weight) is itself influenced by the intervention.^33^ Additionally, seven days of SAAR reduced the respiratory exchange ratio (RER) (Figures 1D and 1E) without any alterations in voluntary wheel running or overall locomotion (Figures 1F, S1G, and S1H).

To test whether the effects on systemic metabolism translate to functional changes, we measured maximal endurance exercise capacity using a one-time treadmill test to exhaustion. Seven days of dietary SAAR significantly increased endurance exercise capacity in sedentary young male mice (Figure 1G), where SAAR mice ran approximately 50% longer than control animals (956.5 ± 306.7 m vs. 634.8 ± 347.3 m, respectively, *p* = 0.009). Increases in running performance was independent of body weight lost during the intervention, as work, calculated by multiplying distance by kg, also showed significant increases after the SAAR intervention compared with control fed animals (Figure 1H).

SAAR is reported to have several sexually dimorphic phenotypes^34^^,^^35^^,^^36^; therefore, we tested whether the running endurance phenotype showed sexual dimorphism. Although young female mice did have lower body weight after SAAR (Figure S1I), they did not increase endurance exercise performance (Figure S1J). Due to this sexually dimorphic response, we exclusively used male mice for subsequent studies.

RER calculates substrate utilization during activity,^37^ and a lower RER indicates increased reliance on fat oxidation. To directly measure substrate utilization during exercise, we performed a one-time endurance exercise test in metabolic treadmills, monitoring RER continuously throughout the exercise bout. Consistent with our non-metabolic treadmill data, SAAR mice ran significantly longer (Figures S1K and S1L). At the terminal spike of RER as the animals reach exhaustion, RER remained lower in SAAR animals, indicating increased reliance on fat oxidation even while nearing exhaustion (Figure 1I).

### Transcriptomics across muscle depots reveal a metabolic shift from glycolytic toward oxidative metabolism after 7 days of feeding

Little is known about the effects of SAAR on metabolic capacity in skeletal muscle. Most work has focused on the role of SAAR in modulating insulin sensitivity and muscle composition or characterized SAARs influence on muscle metabolism after long-term dietary intervention of up to 52 weeks.^9^^,^^38^^,^^39^ Since our data suggested that seven days of SAAR is sufficient to alter systemic metabolism, we aimed to further characterize metabolic changes in specific skeletal muscles.

**Figure 2:**
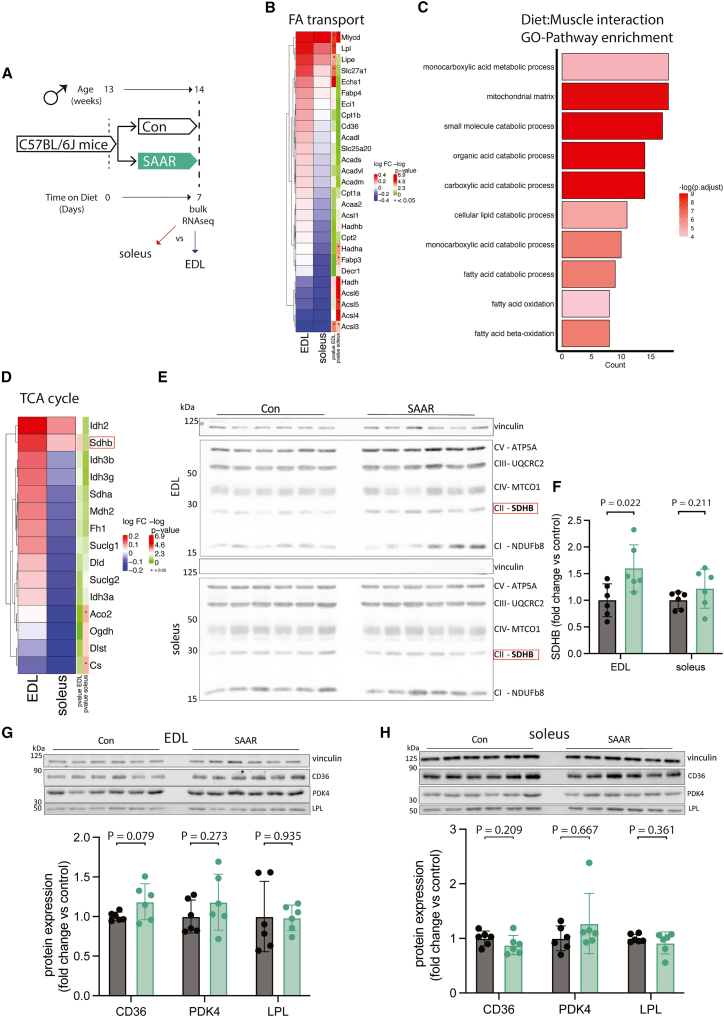
Transcriptomics across muscle depots reveal metabolic shift from glycolytic toward oxidative (A) Experimental set up and color scheme used throughout Figures 2 and S2. (B) Fold changes of transcripts associated with fatty acid (FA) catabolism and transport as identified in supplementary Extended Data Figure 2A in muscle of male mice (*n* = 6) given *ad libitum* access to sulfur amino acid-restricted (SAAR) versus control (Con) diet for seven days. (C) Pathway enrichment analysis of genes showing significant diet-by-muscle interaction effects. (D) Fold changes (SAAR vs. Con) of TCA cycle genes in EDL and soleus. (E) Representative blots of electron transport chain complexes. (F) Quantification of relative protein abundance normalized to vinculin of SDHB for EDL and soleus (*n* = 6) of male mice given *ad libitum* access to SAAR versus Con diet on day seven. (G) Representative blots of CD36, LPL, and PDK4 and vinculin (**top**) and quantification of relative protein abundance normalized to vinculin of CD36, PDK4, and LPL (**bottom**) in EDL (*n* = 6) of male mice given *ad libitum* access to SAAR versus Con diet on day seven. (H) Representative blots of CD36, LPL, and PDK4 and vinculin (**top**) and quantification of relative protein abundance normalized to vinculin of CD36, PDK4, and LPL from blots (**bottom**) in soleus (*n* = 6) of male mice given *ad libitum* access to SAAR versus Con diet on day seven. All panels represent data from mice that were not subjected to endurance running. All data are shown as mean, and error bars indicate SD unless otherwise noted; *p* values indicate the significance of the difference by Student t test between diets; significance is determined by *p* < 0.05. See also Figure S2 and Table S3. Raw data files for Figure 2E are available in the supplement as Data S1 ZIP file.

To investigate SAAR effects on skeletal muscle while accounting for fiber type, we compared glycolytic EDL with oxidative soleus using bulk RNA sequencing (experimental scheme, Figure 2A). Gene set overrepresentation analysis on genes affected by diet independent of muscle type showed upregulation of pathways associated with fatty acid or organic acid catabolism and muscle fiber type switching, and downregulation of pathways associated with extracellular matrix and collagen biosynthesis (Figure S2A). The expression of many genes was coordinated in a fiber-type-dependent fashion (Figures S2B and S2C), so we further explored expression changes after SAAR within EDL and soleus muscles, compared with control diet. Gene-level analysis of fatty acid import and catabolic genes revealed consistently stronger effects in EDL compared with soleus (Figure 2B). A validated ISR/SAAR target gene set,^40^ including the ATF4 target Cth,^41^ revealed minimal muscle-specific differences in response to the dietary treatment (Figures S2F). This indicates that the observed effects are consistent across muscle types, confirming the diet’s efficacy and suggesting that depot-specific responses to SAAR are primarily linked to fatty acid metabolism and independent of the ISR.

We next looked specifically for expression patterns that showed a diet-by-muscle depot interaction. Significant positive interaction terms (enriched in EDL but not soleus after diet) included the previously identified organic acid catabolic processes and β-oxidation, as well as mitochondrial matrix (Figure 2C), suggesting a transcriptomic shift of glycolytic EDL to a more oxidative phenotype. TCA cycle enzymes showed similar muscle depot-specific responses on the transcriptomic level (Figure 2D). We also confirmed transcriptomic changes at the protein level, finding that seven days of SAAR was sufficient to increase electron transport chain (ETC) complexes in both EDL and soleus (Figures 2E, S2D, and S2E). The most pronounced changes were observed in succinate dehydrogenase B (SDHB), which was increased by approximately 50% after SAAR in EDL (Figure 2F), consistent with changes observed at the transcript level.

To determine the overlap between transcriptional adaptation to endurance training and short-term SAAR we compared our data to a recently published exercise training dataset.^42^ Transcriptional regulation of both TCA cycle genes and mitochondrial matrix genes showed overlapping patterns between training and EDL SAAR response, whereas this was not observed to the same extent in the soleus SAAR response (Figures S2G and S2H).

We also assessed changes in protein levels of metabolic enzymes regulating energy homeostasis (CD36, PDK4 and LPL), which were upregulated at the transcriptomic level (Figures 2B, S2C, and S2I), using western blot after seven days of SAAR. CD36 trended toward a significant increase after SAAR in EDL only, consistent with the transcriptomic data. LPL and PDK4 showed non-significant increases after SAAR in EDL (Figure 2G). CD36, LPL, or PDK4 did not show changes after SAAR in soleus (Figure 2H). These changes prompted us to investigate a potential functional role for lipid handling and turnover in the SAAR exercise response.

### SAAR increases muscle lipid flux without altering lipid pool sizes

**Figure 3:**
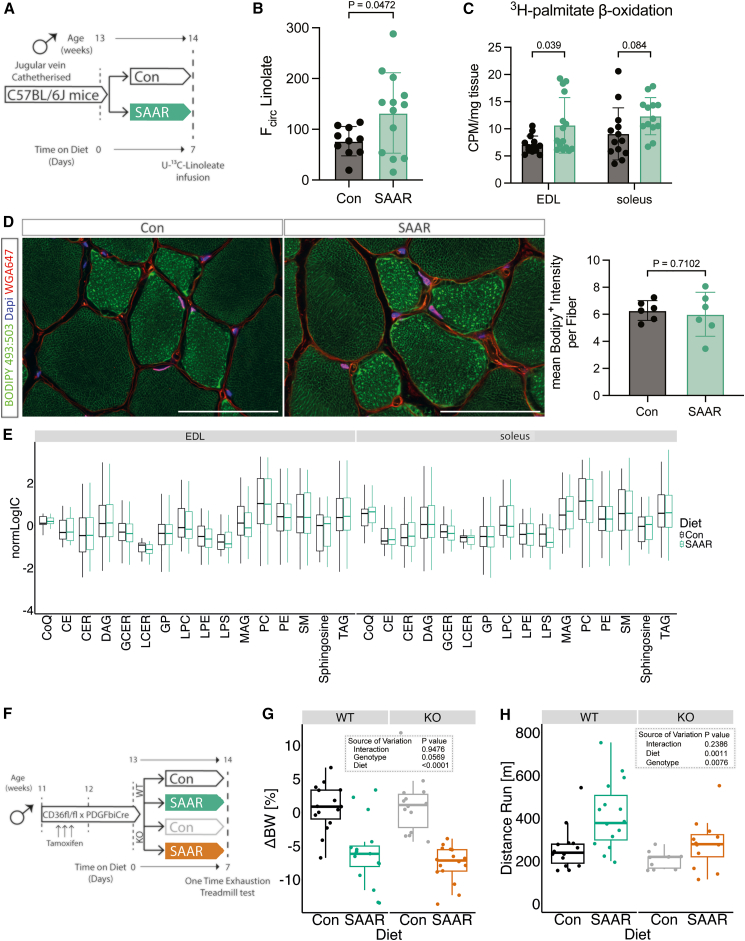
SAAR increases muscle lipid flux without altering lipid pool sizes (A) Experimental design and color scheme used in Figures 3A–3E and S3A. (B) Circulatory carbon flux (*n* = 10–13) of ^13^C_18_-U-Linolate of jugular-vein-catheterized male mice given *ad libitum* access to sulfur amino acid-restricted (SAAR) versus control (Con) diet for seven days. (C) *Ex vivo* β-oxidation measured by incorporation of ^3^H-palmitic acid in ^3^H-H_2_O in muscles of male mice fed a Con or SAAR diet for seven days (*n* = 15). (D) Representative fluorescence images (**left**) of BODIPY 493:503 (green), WGA647 (red), and DAPI (blue) staining in EDL cross sections (scale bar, 50 μm) and quantification of Bodipy^+^ Intensity within fibers (**right**) of male mice fed a Con or SAAR for seven days (*n* = 6). (E) Lipidomics analysis from muscle of male mice fed a Con or SAAR diet for seven days (*n* = 6), summarized as normalized ion counts of each main lipid class. (F) Experimental set up and color scheme used in Figures 3G, 3H, and S3D–S3I. (G) Percent change in body weight (*n* = 16) of male WT and EC^CD36−/−^ mice given *ad libitum* access to SAAR versus Con diet after seven days. (H) Distance ran during a one-time maximal endurance test (n = 9–15) of male WT and EC^CD36−/−^ mice given *ad libitum* access to SAAR versus Con diet on day seven. B–E represent data from mice that were not subjected to endurance running, G and H represent data from mice subjected to maximal endurance testing. All data are shown as mean, and error bars indicate SD unless otherwise noted; *p* values indicate the significance of the difference by Student t test between diets, or two-way ANOVA with Sidak multiple comparisons test between diets and muscle or genotype; significance is determined by *p* < 0.05. See also Figure S3 and Tables S4, S5, and S6.

To test whether the transcript and protein-level changes we observed (Figures 2B–2H) resulted in functional alterations in lipid metabolism, we measured the circulatory turnover flux (F_circ_) of linoleate in pseudo-steady state, as previously validated^43^ in jugular-vein-catheterized mice after seven days of SAAR (experimental scheme, Figure 3A). Linoleate turnover flux was significantly increased after SAAR (Figure 3B). To test whether skeletal muscle fatty acid consumption is involved in driving increased flux, we assessed *ex vivo* skeletal muscle β-oxidation after short-term SAAR using radiolabeled palmitate. Across both EDL and soleus, there was a significant main effect of diet driving increased β-oxidation; however, post hoc tests revealed a significant control vs. SAAR difference in EDL only, highlighting the more pronounced shift of glycolytic muscle to increase lipid oxidation (Figure 3C).

Increased lipid turnover and oxidation can be supported by multiple mechanisms including increased circulating lipid concentrations driving an increase in uptake and storage by mass action^44^ as well as changes in lipid transporter activity. To test if mass action was driving increased turnover and β-oxidation in the muscle, we first measured intramyocellular lipid storage after seven days of diet. Bodipy staining in sections of EDL did not show increased intramyocellular lipid storage between diet groups (Figure 3D). We also performed lipidomic and metabolomic measurements to investigate changes in pool sizes of lipid classes and free fatty acid species after SAAR in tissues. No major lipid classes in either EDL or soleus were affected by seven days of SAAR compared with control (Figure 3E). When assessing differences driven by diet, tissue, or diet-by-tissue interaction across individual lipid species, the dominant source of variance was tissue. However, when focusing on the main effect of diet or diet-by-muscle depot interaction, only a small number (3 and 16, respectively) out of 690 measured lipid species were significantly changed (Table S4). This trend was also generally true for polar metabolites (Table S5). Many metabolites showed significant main effects of muscle depot, for example, carnosine and anserine were enriched in glycolytic tissue as previously reported^45^ (Table S5). We also observed a main effect of diet in multiple metabolites associated with dietary protein restriction (ophthalmic acid^46^) or *Pparg*-associated changes in thermogenesis (aminoisobutyric acid^47^). However, none of these changes showed diet-by-muscle depot interaction effects. Downstream metabolites of the transsulfuration pathway, including taurine, were depleted in the muscle of mice on SAAR (Table S5) suggesting SAAR also affects metabolites downstream of methionine in the muscle. Overall, we did not observe any changes due to diet in the pool size of various free fatty acid species measured in our metabolomic dataset (Figure S3A, Table S5), suggesting that the increased lipid turnover flux is not driven by an increase in intramyocellular lipid availability.

Movement of circulating fatty acids to the skeletal muscle requires their transfer across the EC barrier, with EC CD36 facilitating tissue fatty acid uptake.^30^^,^^31^ Since *Cd36* was upregulated by SAAR specifically in the EDL on both the transcriptomic and protein levels (Figures 2B and 2G–2H), we tested a potential requirement for increased lipid shuttling into the muscle via EC CD36. To explore this hypothesis, we generated a tamoxifen-inducible EC-specific CD36 knockout (KO) by crossing mice with a *Cd36* floxed allele with mice hemizygously expressing PDGFβiCre (EC^CD36−/−^).^48^ Cre^+^ and Cre^−^ littermates were distributed equally between control and SAAR groups (experimental scheme, Figure 3F). One cohort was used to study metabolic phenotypes, and another was used to perform a one-time endurance exercise test. The KO efficiency was confirmed by flow cytometry, gating for CD31^+^CD36^+^ populations in both skeletal muscle and BAT (Figures S3B–S3E). EC *Cd36* KO did not affect the body weight changes induced by SAAR (Figures 3G and S3F). In the endurance exercise test, SAAR significantly increased running performance in wild-type (WT) animals only. Post hoc testing showed increased running performance after SAAR in WT animals, with EC *Cd36* deletion attenuating the running performance increase (Figure 3H). To test whether EC *Cd36* deletion had an effect on fatty acid composition in skeletal muscle and circulation after one week of diet, we performed bulk metabolomics. After false discovery rate correction, very few metabolites were significantly affected by genotype or diet in EDL (13 and 7, respectively), soleus (3 and 3 respectively), or serum (16 and 28, respectively), none of which were consistent across the three tissues (Figures 3G and 3H; Table S6).

### FGF21 is dispensable for increased running capacity upon SAAR

**Figure 4:**
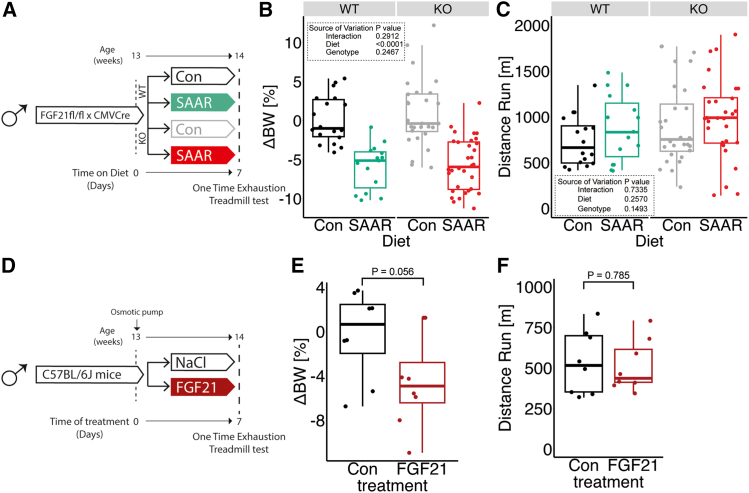
FGF21 is dispensable for increased running capacity upon SAAR (A) Experimental design and color scheme used in Figures 4B, 4C, and S4A–S4C. (B) Percent change in body weight (*n* = 15–32) of male WT or FGF21KO mice given *ad libitum* access to sulfur amino acid-restricted (SAAR) versus control (Con) diet for seven days. (C) Distance ran during a one-time maximal endurance test (*n* = 15–29) of male WT or FGF21KO mice given *ad libitum* access to SAAR versus Con diet on day seven. (D) Experimental set up and color scheme used throughout E and F and Figure S4D and S4E. (E) Percent change in body weight (*n* = 8) of NaCl or recombinant FGF21-treated male mice for seven days. (F) Distance ran during a one-time maximal endurance test (*n* = 8) of NaCl or recombinant FGF21-treated male mice on day seven. B represents data from mice that were not subjected to endurance running; C, E, and F represent data from mice subjected to maximal endurance testing. All data are shown as mean, and error bars indicate SD unless otherwise noted; *p* values indicate the significance of the difference by Student t test between treatments, or two-way ANOVA with Sidak multiple comparisons test between diets and genotype; significance is determined by *p* < 0.05. See also Figure S4.

Given that FGF21 is a well-established endocrine mediator of systemic metabolic responses to SAAR, including increased lipid oxidation and EE,^6^^,^^35^^,^^49^^,^^50^ we investigated whether it is necessary for the enhanced running performance observed in our model. While our primary focus was on skeletal muscle adaptation, assessing FGF21 allowed us to determine whether this canonical SAAR-induced hepatokine contributes directly to exercise capacity. To test whether FGF21 is required for increased running performance upon SAAR, mice with an *Fgf21* floxed allele were crossed with CMV-Cre to achieve stable whole-body *Fgf21* knockout (FGF21KO) (experimental scheme, Figure 4A). FGF21KO and WT littermates were evenly distributed between dietary groups with one cohort used to assess metabolic changes and a second cohort for one time treadmill testing after seven days of diet. No significant effects of genotype on SAAR-induced body weight changes were observed (Figures 4B and S4A). However, in agreement with previous observations, the decreased body weight in FGF21KO animals on SAAR can be mainly explained by a decrease in food intake^35^ (Figure S4B). FGF21KO had no effect on running performance, with both genotypes tending to increase after SAAR (Figure 4C). However, variability in the control groups limited these trends from reaching statistical significance in either genotype (Figure 4C). KO efficiency was confirmed by serum FGF21 ELISA, where SAAR increased circulating FGF21 levels, and no FGF21 was detected in serum of FGF21KO animals (Figure S4C).

We, and others, have observed increases in hepatic FGF21 expression and serum levels after SAAR^35^^,^^49^^,^^50^; therefore, we tested whether FGF21 alone is sufficient to increase running performance by infusing recombinant FGF21 for seven days. We implanted WT C57BL/6J male mice with osmotic minipumps loaded with either saline solution or recombinant FGF21 (dosed at 1 mg/kg/day) for seven days (experimental scheme, Figure 4D). Expression of known FGF21-responsive genes in BAT, a positive control due to its high sensitivity to FGF21, was measured by qPCR to confirm induction of FGF21 signaling. Both *Ucp1* and *Fgf21* were upregulated in the exogenously supplied FGF21 group as well as the SAAR group (Figure S4E). Exogenous FGF21 caused a significant reduction in body weight, although to a lesser extent than what we observe with the SAAR diet (Figures 4E and S4D). On day seven of the intervention the animals underwent a one-time treadmill test. We observed that exogenous FGF21 administration was not sufficient to mimic the increased endurance exercise capacity seen in SAAR-fed mice (Figure 4F). Taken together these data suggest that FGF21 is not necessary for increased endurance exercise capacity after SAAR nor is it sufficient to increase endurance exercise capacity on its own.

### Inhibition of VEGFR signaling blocks increased endurance exercise capacity by SAAR

**Figure 5:**
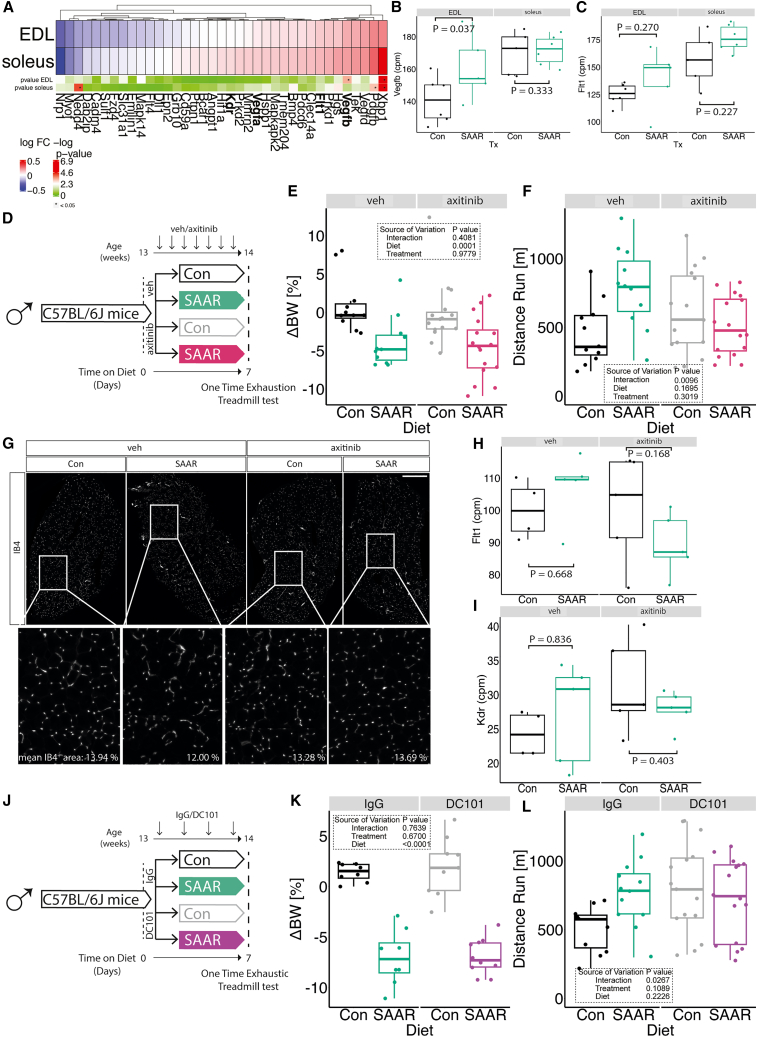
Inhibition of VEGFR signaling blocks increased endurance exercise capacity by SAAR (A) Fold changes of transcripts associated with Vegf signaling using transcriptomic dataset presented in Figure 2 in muscles of male mice (*n* = 6) after sulfur amino acid restriction (SAAR) compared with control (Con) diet for seven days. (B) Normalized count values of Vegfb in EDL and soleus from bulk RNA sequencing (*n* = 6) of male mice given *ad libitum* access to SAAR versus Con diet on day seven using transcriptomic dataset presented in Figure 2. (C) Normalized count values of Flt1 in EDL and soleus from bulk RNA sequencing (*n* = 6) of male mice given *ad libitum* access to SAAR versus Con diet on day seven using transcriptomic dataset presented in Figure 2. (D) Experimental set up and color scheme used in Figures 5E, 5F, S5C, and S5D. (E) Percent change in body weight (*n* = 16–24) of male mice treated with vehicle (veh) or axitinib via oral gavage in combination with *ad libitum* access to SAAR versus Con diet after seven days. (F) Distance ran during a one-time maximal endurance test (*n* = 16–24) of male mice treated with veh or axitinib via oral gavage in combination with *ad libitum* access to SAAR versus Con diet for seven days. (G) Representative fluorescence images of IB4 (white) staining in EDL cross sections of mice fed a Con or SAAR Diet, co-treated with veh or axitinib via oral gavage (scale bar, 400 μm) for seven days (n = 5–8). (H) Normalized count values of Flt1 in EDL treated with either veh or axitinib from bulk RNA sequencing (*n* = 5) of male mice given *ad libitum* access to SAAR versus Con diet on day seven. (I) Normalized count values of Kdr in EDL treated with either veh or axitinib from bulk RNA sequencing (*n* = 5) of male mice given *ad libitum* access to SAAR versus Con diet on day seven. (J) Experimental set up and color scheme used in Figures 5K, 5L, S5J, and S5K. (K) Percent change in body weight (n = 8–10) of male mice treated with IgG or DC101 via i.p. injection every other day in combination with *ad libitum* access to SAAR versus Con diet after seven days. (L) Distance ran during a one-time maximal endurance test (*n* = 12–16) of male mice treated with IgG or DC101 via i.p. injection every other day in combination with *ad libitum* access to SAAR versus Con diet on day seven. A-C, E, G-I, represent data from mice that were not subjected to endurance running; F, K, and L represent data from mice subjected to maximal endurance testing. All data are shown as mean, and error bars indicate SD unless otherwise noted; *p* values indicate the significance of the difference by two-way ANOVA with Sidak multiple comparisons test between diets and treatment; significance is determined by *p* < 0.05. See also Figure S5 and Table S7.

Our previous work demonstrated that long-term SAAR increases EC VEGF levels and induces angiogenesis in the muscle,^11^ which is a known adaptation that facilitates exercise performance.^51^^,^^52^ We therefore examined the transcriptomic signature associated with VEGF-signaling in our bulk transcriptomic datasets. Interestingly, we observed that *Vegfb* was increased by SAAR specifically in the EDL (Figures 5A and 5B) and *Flt1* (encoding VEGFR1) trended toward an increase in both EDL and soleus (Figures 5A and 5C). *Vegfa* and *Kdr* (encoding VEGFR2) were not affected at the transcript level by short-term SAAR (Figures S5A and S5B). To test whether induction of VEGF signaling was a driver of increased running capacity, we treated animals with the pan-VEGFR inhibitor axitinib or vehicle control by oral gavage throughout the seven-day SAAR dietary intervention (experimental scheme, Figure 5D). Treatment with axitinib did not affect the body weight response to SAAR (Figures 5E and S5C). However, upon one-time endurance exercise testing at day seven, axitinib blunted the increased running performance of SAAR mice (Figure 5F). To test whether this was due to the prevention of SAAR-induced angiogenesis we measured vascular density in the muscle. Immunofluorescent labeling of the vasculature in cryosections of EDL did not show any significant changes in vascular area as a function of either SAAR or axitinib treatment (Figures 5G and S5D). Labeling proliferating ECs using EdU also did not show an effect of SAAR on EC proliferation in either EDL or soleus after seven days (Figure S5E). Similarly, quantifying total CD31^+^ cells by flow cytometry revealed no increase in EC number after seven days of SAAR in either muscle or BAT (Figures S5F and S5G).

Since angiogenesis was not changed upon seven days of axitinib treatment or short-term SAAR, we next assessed whether transcriptional regulation of the VEGF/VEGFR genes were altered using RNA sequencing of EDL from mice treated with axitinib during seven days of SAAR. While axitinib treatment did not strongly affect expression of *Vegfa* or *Vegfb*, axitinib co-treatment did block the SAAR-induced increase in the receptors *Flt1* and *Kdr* (Figures 5H and 5I and Extended Data Figures 5H and 5I). Interestingly, we also observed that inhibition of VEGF signaling downregulated fatty acid transport-associated transcripts (Figure S5J) but on the other hand further promoted SAAR-induced increases in ETC-associated genes (Figure S5K), suggesting that VEGF signaling may control fatty acid availability but not oxidation capacity.

To differentiate between the different VEGFRs and their contribution to increased endurance exercise capacity, we treated mice with DC101, a VEGFR2-specific neutralizing antibody^53^ or an IgG control and measured metabolic as well as exercise parameters (experimental scheme, Figure 5J). Inhibiting VEGFR2 specifically with DC101 did not alter the dietary effect of SAAR on either reducing body weight or increasing food intake (Figures 5K, S5L, and S5M). However, inhibiting VEGFR2 specifically attenuated the SAAR-induced increase in endurance exercise capacity compared with control diet mice treated with DC101 (Figure 5L). While SAAR still elevated endurance capacity compared with baseline in the IgG-treated group, the additional boost typically seen with SAAR was blunted by VEGFR2 inhibition.

## Discussion

Using healthy sedentary male mice, we report that short-term dietary SAAR rewires systemic and muscle metabolism and increases endurance exercise capacity potentially through angiogenesis-independent VEGF-signaling pathways.

We found that seven days of SAAR promoted a metabolic shift toward whole-body fat oxidation during rest as well as exercise. We further showed increased whole-body linoleate F_circ_ and β-oxidation in skeletal muscle. This switch was reflected in increased expression of genes related to fat oxidation and oxidative phosphorylation in skeletal muscle. The activation of fat metabolism was more pronounced in muscle with a higher proportion of glycolytic fibers, such as the EDL. However, we did observe increases in oxidative phosphorylation genes in more oxidative muscles as well, and some pathways associated with increased fatty acid catabolism and organic acid import were increased upon diet rather than being specific for diet-muscle interaction. We thus hypothesize that changes in EDL are more prone to elicit relevant changes on a functional level due to the low baseline levels of these processes.

Seven days of SAAR was sufficient to increase both circulatory fatty acid turnover and muscle-specific β-oxidation. We did not observe changes in intramyocellular lipid storage, lipid composition, or free fatty acid pool size upon SAAR. This suggests that the increase in fat oxidation was fueled by increased fatty acid uptake from the circulation, which requires transendothelial transport. Consistently, expression of the fatty acid transporter CD36 was increased upon SAAR at both the RNA and protein levels. Restricting the acute supply of fatty acid influx via endothelial CD36 deletion during exercise attenuated the endurance exercise capacity phenotype. This underscores the necessity (but not necessarily sufficiency) of CD36-mediated fatty acid supply for SAAR to elicit its beneficial effects on endurance exercise capacity. While increased lipid uptake upon SAAR is required to fuel oxidative phosphorylation, whether increased oxidative phosphorylation machinery is also required remains to be tested.

An important consideration is the potential for glucose limitation in the context of reduced fatty acid availability. Since SAAR has been shown to reduce circulating glucose concentrations and tissue glycogen stores, the absence of endothelial CD36 could conceivably increase reliance on glucose during exercise, leading to faster depletion of glucose availability and possibly hypoglycemia, thereby impairing endurance. Although we did not directly assess glucose parameters (e.g., blood glucose or muscle glycogen) during the exercise test, we recognize this as a key limitation. Future studies incorporating metabolic flux analysis or *in vivo* glucose measurements during exercise would be valuable to clarify the contribution of glucose depletion to the impaired endurance phenotype observed in EC^CD36−/−^ mice.

Previous work has demonstrated that 18 weeks of SAAR in aged mice is sufficient to rescue age-associated lean mass losses and also to prevent muscle hypertrophy after overload.^9^ Running performance or other indicators of muscle fitness were not assessed; the authors did show elevated levels of succinate dehydrogenase activity suggesting increased oxidative capacity after SAAR in skeletal muscle, consistent with our findings. Similar findings on citrate synthase activity and mitochondrial biogenesis have also been observed.^10^ Functionally, it is widely established that SAAR increases EE^6^^,^^7^^,^^10^^,^^35^^,^^36^; however, whole-body substrate utilization or functional performance capacity has been less well studied. Increases in total home-cage activity were measured after 8 weeks of SAAR feeding, where studies^36^^,^^54^ reported lowered RER after 5 weeks of a western diet restricted in sulfur amino acids but these data could be influenced by different lipid compositions of the diet.

It has been well established that MR induces significant metabolic remodeling across many tissues.^7^^,^^10^^,^^35^^,^^55^^,^^56^^,^^57^^,^^58^^,^^59^ Fang et al.^55^ provided a comprehensive overview, highlighting that MR leads to decreased hepatic *de novo* lipogenesis and triglyceride synthesis,^58^ while simultaneously enhancing lipogenic and oxidative capacities in WAT.^57^ These adaptations contribute to improved systemic metabolic flexibility and insulin sensitivity.^56^ Notably, while substantial transcriptional changes occur in the liver and WAT, skeletal muscle exhibits minimal transcriptional response to MR.^10^ Our findings build upon this knowledge by demonstrating that short-term SAAR can rapidly enhance endurance capacity and promote a metabolic shift toward increased fatty acid oxidation in skeletal muscle. This suggests that, despite limited transcriptional changes, skeletal muscle undergoes functional metabolic adaptations in response to SAAR, potentially mediated by post-transcriptional mechanisms or inter-tissue signaling pathways. These insights underscore the complexity of tissue-specific responses to amino acid restriction and highlight the need for further investigation into the underlying mechanisms facilitating these adaptations.

It was previously shown that long-term SAAR drives angiogenesis in skeletal muscle via increased VEGF signaling in ECs.^11^ In this study, one week of SAAR was not sufficient to stimulate neovascularization in the muscle, which is consistent with the 2- to 3-week timeline for neovascularization following exercise training.^60^ We therefore propose that the improved endurance exercise capacity upon SAAR is driven by a metabolic shift favoring fatty acid transport by ECs and increased oxidation by skeletal muscle rather than by increased muscle vascularization. Interestingly, pan VEGFR inhibition or specific VEGFR2 inhibition also inhibited the endurance exercise capacity increase upon SAAR. This finding implicates VEGF signaling in SAAR’s endurance exercise effects independent of its most prominent role in angiogenesis. However, we acknowledge that not all canonical actions of VEGF were assessed in this study and delineating which VEGF is responsible for induced exercise capacity was not accomplished. VEGF is also known to promote vasodilation and vascular permeability, which can facilitate nutrient and substrate delivery to muscle tissue.^61^^,^^62^ These functions may have contributed to enhanced lipid delivery and utilization during exercise following SAAR, and further studies will be needed to disentangle these roles from those related to angiogenesis or metabolic signaling. Notably, DC101 treatment (the specific VEGFR2 antibody used) also increased running distance in control-diet mice, indicating that VEGFR2 inhibition exerts diet-independent effects on exercise performance. This complicates the interpretation that VEGFR2 exclusively mediates the SAAR endurance phenotype. Nevertheless, the relative enhancement in endurance capacity was attenuated under SAAR conditions compared with vehicle, supporting a contributory role for VEGFR2 signaling.

While our study focused primarily on VEGF signaling and substrate metabolism, the transcriptomic dataset suggests that Xbp1 may contribute to the adaptive response of skeletal muscle to SAAR. Xbp1 is a central effector of the unfolded protein response, activated in response to ER stress, and has been shown to regulate mitochondrial function, cellular metabolism, and muscle regeneration. Recent work by Joshi et al.^63^ demonstrated that myofiber-specific Xbp1 is essential for coordinating regenerative myogenesis, influencing both myogenic and stromal cell populations. The increase in Xbp1 expression we report may therefore reflect a compensatory mechanism to maintain proteostasis and cellular resilience under amino-acid-restricted conditions. Given the energetic demands associated with enhanced lipid oxidation and endurance capacity induced by SAAR, it is plausible that Xbp1 supports mitochondrial or metabolic remodeling required to sustain this phenotype. Although we did not explore this pathway in mechanistic detail, these findings raise the possibility that Xbp1 contributes to the broader transcriptional and functional adaptations of skeletal muscle under SAAR. Future studies are needed to determine whether Xbp1 plays a causal role in mediating the metabolic and performance benefits of SAAR.

### Limitations of the study

Sexual dimorphism in response to SAAR has been consistently observed across multiple studies,^6^^,^^34^^,^^36^^,^^64^ and our findings are in line with this body of work. In the current study, we focused exclusively on male mice after preliminary data indicated that SAAR did not improve exercise capacity in females. While a comprehensive investigation into the mechanisms underlying this sex-specific effect is beyond the scope of the present study, one plausible explanation involves the differential regulation of endogenous FGF21 and its downstream responses.^34^ Among these is VEGF signaling, which has been shown to be upregulated by SAAR through an FGF21-ATF4-dependent axis.^11^ In our study, we found that exogenous administration of FGF21, despite inducing classical FGF21-responsive pathways such as thermogenesis and weight loss, was not sufficient to increase endurance exercise capacity (Figures 4E and 4F), suggesting that context-specific endogenous FGF21 signaling, potentially in combination with other diet-induced cues, is critical for the exercise phenotype. A reduced endogenous FGF21 response in females may therefore blunt VEGF-mediated adaptations and partially explain the lack of improvement in endurance. We acknowledge that the exclusion of female mice limits the translatability of our findings, and future studies should aim to clarify the hormonal and molecular pathways contributing to the sex-specific effects of SAAR, as well as the tissue-specific roles of FGF21 to assess the contribution to the systemic metabolic adaptations in non-muscle tissues.

An important consideration in interpreting the metabolic effects of SAAR is the intertwined biology of methionine and cysteine. While our study employed a combined restriction of both sulfur-containing amino acids, recent work^65^^,^^66^ demonstrated that the metabolic consequences of dietary cysteine restriction, such as reduced glutathione and CoA levels, FGF21 induction, and weight loss, only became apparent when methionine-to-cysteine conversion was genetically blocked via Cth deletion. This highlights the difficulty in fully disentangling the individual contributions of methionine and cysteine using standard dietary models. Given this metabolic interdependence, it remains possible that cysteine restriction independently contributes to the observed adaptations. Future studies using diets that restrict methionine alone or in combination with genetic models that uncouple sulfur amino acid metabolism will be essential to delineate the specific roles of each amino acid.

Here we employed a fixed running of 20 m/min for endurance tests in both control and SAAR-fed mice. We did not assess VO_2max_ or maximal running speed to determine relative exercise intensity. Therefore, we cannot rule out the possibility that SAAR mice ran at a lower relative intensity, which could partially explain their increased time to exhaustion. Nevertheless, the ability to sustain a fixed workload for a longer duration remains physiologically meaningful and suggests improved metabolic efficiency. Future studies using graded exercise testing or VO_2max_ assessments are warranted to better characterize the relative intensity and endurance phenotype.

Due to technical limitations, we were unable to unravel the specific contribution of VEGF-A versus -B as the molecular driver of endurance exercise capacity increases after short-term SAAR. We found that both VEGF-A and VEGF-B are transcriptionally upregulated after dietary SAAR. Inhibition of VEGFR2, which exclusively binds VEGF-A, prevented increased running performance, suggesting a causal role for VEGF-A and its receptor. Skeletal muscle VEGF has been reported to be crucial for exercise training, as deletion of VEGF blunted exercise capacity in mice.^51^ We and others have previously shown that SAAR increases endothelial VEGF signaling.^11^^,^^67^ However, an increase in VEGF-B could still push increased VEGF-A/VEGFR2 interaction due to competition for VEGFR1 binding. Further research and genetic models will be required to completely address this question. Moreover, since axitinib is a broad-spectrum tyrosine kinase inhibitor with known off-targets, we cannot exclude that receptor tyrosine kinases beyond VEGFRs may contribute to the observed effects. This limitation should be considered when interpreting our findings, and future studies with more specific tools will be necessary to define the precise role of VEGFR signaling in the SAAR-induced exercise phenotype.

We were also unable to directly measure fatty acid uptake in muscle tissue due to technical limitations. Oral gavage of radiolabeled fatty acids resulted in minimal incorporation into distal skeletal muscles such as the EDL and soleus. As such our conclusions are based on whole-body estimates of fatty acid utilization, derived from the fractional turnover rate (Fcirc) of stably labeled linoleic acid. The upregulation of oxidative machinery suggests that fatty acids are rapidly oxidized upon uptake rather than stored, supporting a high-throughput, low-reserve metabolic state. Future studies employing stable isotope tracers will be important to better resolve lipid kinetics and confirm this dynamic metabolic shift *in vivo*.

In summary, we report that one week of dietary restriction of the sulfur-containing amino acids methionine and cysteine (provided in the diet as cystine) is sufficient to induce an improvement in endurance exercise capacity in young sedentary male mice. We highlight a potential role for angiogenesis-independent VEGF signaling and EC CD36-dependent fatty acid transport in this phenotype by influencing muscle substrate availability.

## Resource availability

### Lead contact

Requests for further information and resources should be directed to and will be fulfilled by the lead contact, Sarah Mitchell, PhD (sm3272@princeton.edu).

### Materials availability

This study did not generate new materials.

### Data and code availability

All data related to the bulk RNA sequencing for soleus and extensor digitorum longus muscle from mice fed either control or sulfur amino acid-restricted (SAAR) diet for 7 days are available at: https://www.ncbi.nlm.nih.gov/bioproject/1236535.

All other relevant data are available from the corresponding author on request.

RNA sequencing data from the extensor digitorum longus muscle of mice subjected to either a control diet or a sulfur amino acid-restricted (SAAR) diet for seven days with or without axitinib are available at: http://www.ncbi.nlm.nih.gov/bioproject/1237784.

Metabolomics and lipidomics data have been deposited at Metabolomics Workbench (https://www.metabolomicsworkbench.org/). Identifier for data is https://doi.org/10.21228/M8BR77 release date 16 April 2025.

No unique code was generated for this study.

## Acknowledgments

This work was supported by the 10.13039/100000049National Institute on Aging (P01AG055369 to S.J.M. and J.R.M.), an ETH Zurich Doc. Mobility Fellowship (C.G.M.) and 10.13039/501100003006ETH Zurich core funding. W.L. is supported by the 10.13039/100000054National Cancer Institute (R50CA211437). We want to thank the center of PhenoGenomics at EPFL for helping us perform the metabolic running experiments. We dedicate this work to our late mentor and friend, J.R.M.

## Author contributions

Conceptualization, C.G.M., M.R.M., K.D.B., J.R.M., and S.J.M.; methodology, C.G.M., M.R.M., J.Z., S.G., J.E.A., W.L., C.J.H., and T.A.; resources, A.L., F.A., J.D.R., K.D.B., and S.J.M.; writing, C.G.M., M.R.M., K.D.B., and S.J.M.; funding acquisition, C.G.M., M.R.M., and S.J.M.

## Declaration of interests

J.D.R. is a member of the Rutgers Cancer Institute of New Jersey (RCINJ) and the University of Pennsylvania Diabetes Research Center (U Penn DRC); a director of the U Penn DRC-Princeton inter-institutional metabolomics core and RCINJ metabolomics core; an advisor and stockholder in Colorado Research Partners, Bantam Pharmaceuticals, Barer Institute, Rafael Pharmaceuticals, Empress Therapeutics, and Marea Therapeutics; a founder, director, and stockholder of Farber Parnters, Raze Therapeutics; a founder of, advisor to, and stockholder in Marea Therapeutics and Fargo Biotechnologies; and an inventor of patents held by Princeton University.

## STAR★Methods

### Key resources table

**Table:** 

REAGENT or RESOURCE	SOURCE	IDENTIFIER
**Antibodies**		

Goat anti-Mouse/Rat CD31/PECAM-1 antibody	R&D Systems	Cat# 3628; RRID: AB_2161028
Rat anti-CD31 antibody	Abcam	Cat# ab7388; RRID: AB_305905
PE Rat Anti-Mouse CD31	BD Biosciences	Cat# 553373; RRID: AB_394815
PerCP Rat Anti-Mouse CD45	BD Biosciences	Cat# 557235; RRID: AB_10642171
Mouse (IgG2b) Myosin Heavy Chain Type I antibody (BA-F8)	DSHB	Cat# BA-F8; RRID: AB_10572253
Mouse (IgG1) Myosin Heavy Chain Type IIA antibody (SC-71)	DSHB	Cat# SC-71; RRID: AB_2147165
Mouse (IgM) Myosin Heavy Chain Type IIB antibody (BF-F3)	DSHB	Cat# BF-F3; RRID: AB_2266724
Total OXPHOS Rodent WB Antibody Cocktail	Abcam	Cat# ab110413; RRID: AB_2629281
PE Hamster Anti-Mouse CD36	BioLegend	Cat# 102605; RRID: AB_389349
Rabbit anti-CD36 antibody	Abcam	Cat# ab124515; RRID: AB_2924667
Rabbit anti-PDK4 antibody	Abcam	Cat# ab214938; RRID: AB_2864318
Goat anti Human/Mouse Lipoprotein lipase antibody	R&D Systems	Cat# AF7197; RRID: AB_10972480
Wheat Germ Agglutinin (WGA)	Thermo Fisher Scientific	Cat# W11262
IRDye 800RD Goat anti-Mouse IgG (H + L)	LI-COR BioScience	Cat# LIC-926-68070
IRDye 680RD Goat anti-Rabbit IgG (H + L) Highly Cross-Adsorbed	LI-COR BioScience	Cat# LIC-926-68071
Anti-rabbit IgG, HRP-linked	Cell Signaling	Cat# 7074
Goat anti-Mouse IgG2b Cross-Adsorbed Secondary Antibody, Alexa Fluor™ 488	Thermo Fisher Scientific	Cat# A-21141
Goat anti-Mouse IgG1 Cross-Adsorbed Secondary Antibody, Alexa Fluor™ 350	Thermo Fisher Scientific	Cat# A-211120
Goat anti-Mouse IgGM (Heavy Chain) Cross-Adsorbed Secondary Antibody, Alexa Fluor™ 568	Thermo Fisher Scientific	Cat# A-21043

**Chemicals, peptides, and recombinant proteins**		

Collagenase IV	Thermo Fisher Scientific	Cat# 17104019
Dispase II	Sigma-Aldrich	Cat# D4693
Hoechst	Thermo Fisher Scientific	Cat# 62249
5-Ethynyl-2′-deoxyuridine (EdU)	Thermo Fisher Scientific	Cat# C10632/4
Alexa 647 Fluor conjugated isolectin B4	Thermo Fisher Scientific	Cat# I32450
Tamoxifen	Sigma-Aldrich	Cat# T5648
Axitinib	MedChemExpress	Cat# HY-10065
InVivoMAb rat IgG1 isotype control	BIOZOL	Cat# BXC-BE0088
*InVivo* MAb anti Mouse VEGFR- 2, Clone: [DC101], Rat, Monoclonal	BIOZOL	Cat# BXC-BE0060
Linoleic Acid (18:2), sodium salt (U-^13^C_18_, 98%)	Cambridge Isotope Laboratories	Cat# CLM-10487-PK
Palmitic Acid, [9,10-^3^H(N)]-, 1 mCi, (37MBq)	Perkin Elmer	Cat# NET043001MC
BODIPY™ 493/503	Thermo Fisher Scientific	Cat# D2191
mouse recombinant FGF21	Preprotech	Cat# 450-56

**Critical commercial assays**		

Pierce BCA Protein Assay Kit	Thermo Fisher Scientific	Cat# 23225
mVEGF Elisa	R&D Systems	Cat# MMV00
mVEGFB Elisa	Abcam	Cat# ab289700
mFGF21 Elisa	R&D Systems	Cat# MF2100
RNeasy Kit	QIAGEN	Cat# 74034
High Capacity cDNA Reverse Transcription Kit	Thermo Fisher Scientific	Cat# 43-688-13
SYBRGreen-based Master Mix	Thermo Fisher Scientific	Cat# A25778
Click-iT Cell Reaction Buffer Kit	Thermo Fisher Scientific	Cat# C10269

**Deposited data**		

Muscle transcriptomics raw and analyzed	This paper	Tables S3 and S7 https://www.ncbi.nlm.nih.gov/bioproject/1236535 and http://www.ncbi.nlm.nih.gov/bioproject/1237784
Muscle lipidomics	This paper	Table S4 Metabolomics Workbench Project https://doi.org/10.21228/M8BR77
Muscle and plasma metabolomics from WT and WT and CD36^EC−/−^ mice	This paper	Tables S5 and S6 Metabolomics Workbench Project https://doi.org/10.21228/M8BR77

**Experimental models: Organisms/strains**		

Mouse: C57BL/6J wild type	Charles River Laboratories	N/A
Mouse: *CD36*^fl/fl^	Charles River Laboratories	N/A
Mouse: *fgf21*^*fl/fl*^	Charles River Laboratories	N/A
Mouse: *pdgfb*-Cre^ERT2^	Claxton et al.^48^	N/A

**Oligonucleotides**		

*Fgf21*: F: CAAATCCTGGGTGTCAAAGC	N/A	N/A
*Fgf21*: R: CATGGGCTTCAGACTGGTAC	N/A	N/A
*Ucp1*: F: GCATTCAGAGGCAAATCAGC	N/A	N/A
*Ucp1*: R: GCCACACCTCCAGTCATTAAG	N/A	N/A
*18s:* F CATGCAGAACCCACGACAGTA	N/A	N/A
*18s:* R CCTCACGCAGCTTGTTGTCTA	N/A	N/A
*Actin:* F AGCTTCTTTGCAGCTCCTTCGTTG	N/A	N/A
*Actin:* R TTCTGACCCATTCCCACCATCACA	N/A	N/A

**Software and algorithms**		

FlowJo Software (version 10.4.2)	Three Star	https://www.flowjo.com/
ImageJ (for image analysis)	NIH	https://imagej.nih.gov/ij/
Prism 8 (version 8.0.0)	GraphPad Software	https://www.graphpad.com/scientific-software/prism/
Adobe Illustrator CS6 (version 16.0.4)	Adobe	https://www.adobe.com/
R 4.0.3	cran.r-project	https://cran.r-project.org/bin/windows/base/
El Maven	Elucidata	https://github.com/ElucidataInc/ElMaven/releases

### Experimental models and study details

#### Mice

All animal experiments were approved by the local animal ethics committees (Kantonales Veterinäramt Zürich, licenses ZH211/19, ZH149/21, ZH133/23, Animal Care and Use Committee for Princeton University, Harvard Medical Area Institutional Animal Care and Use Committee (IACUC) and Service de la Consommation et des Affaires Vétérinaires SCAV-EXPANIM, license VD-3664), and performed according to local guidelines (TschV, Zurich) and the Swiss animal protection law (TschG). Health status of all mouse lines was regularly monitored according to FELASA guidelines. Mice used in experiments were 10–14 weeks old. Mice were housed in standard housing conditions (22°C, 12 h light/dark cycle). Mice purchased from a vendor were allowed to acclimate in the facility for at least 10 days before starting a study. Prior to the start of studies mice were given *ad libitum* (AL) access to chow diet (18% protein, 4.5% fat, #3437, Provimi Kliba SA) and water. During studies mice were given AL access to water and the respective study diet.

Experimental diets were based on Research Diets D12450J with approximately 18% of calories from protein, 10% from fat and 72% from carbohydrates. SAAR diets containing 1.15g methionine/kg food and lacking cysteine^1^ were provided AL. Food intake was monitored daily during experiments. The Research Diets product number for the control diet is A17101101 and for SAAR diet is A17101103. Detailed descriptions of both diets are provided in Table S1.

Wild type (WT) C57BL/6J and *Cd36 LoxP*/*LoxP* mice (*Cd36*^*tm1.1Ijg*^/J) were purchased from Charles River (Freiburg im Breisgau, Germany). To obtain inducible endothelial cell-specific Cd36 knockout (EC^CD36−/-^) mice, *Cd36 LoxP*/*LoxP* mice were crossed with *PDGFβ.iCreER* mice, an EC-selective inducible Cre-driver line.^48^ Recombination was induced in 8–10 weeks old male mice by daily intraperitoneal (i.p.) administration of 1 mg tamoxifen (T5648, Sigma-Aldrich) dissolved in 1:10 ethanol:corn oil solution for 3 consecutive days. A wash out period of at least seven days was allowed before starting the experiments. Tamoxifen-treated Cre-negative littermates were used as control for all experiments. *Fgf21* knockout (*Fgf21*KO) mice were generated by crossing *Fgf21*^*LoxP*/*LoxP*^ mice (B6.129S6(SJL)-Fgf21tm1.2Djm/J) with *loxP* sites flanking exons 1–3 of the Fgf21 gene with CMV-Cre expressing mice (B6.C-Tg(CMV-Cre)1Cgn/J). The resulting offspring had a deletion in exons 1–3 of *Fgf21* in all tissues. The line was subsequently maintained by breeding animals heterozygous for the deletion allele.

Mouse recombinant FGF21 (Cat# 450-56, Peprotech) was dissolved and diluted in sterile distilled water to a final dosage of 1 mg/kg/day. The filled 1007D Alzet osmotic minipump was pre-soaked for 24 h in NaCl at 37°C in a dry incubator. Mice were anesthetized with 3% isoflurane in 2 L O_2_ and kept at 37°C with an electrical heating pad. A 1-cm incision was made in the skin of the upper back/neck to implant the sterile, preloaded minipump. 5-0 Prolene surgical suture was used to close the wound. Mice received paracetamol (2 mg/ml Dafalgan, UPSA) in the drinking water for 48 h postoperatively.

Aseptic surgery was performed to place catheters in the right jugular vein connected to a vascular access button implanted under the skin on the back of the mouse (Instech Laboratories). Mice were allowed to recover from catheterization surgery for at least 5 days before experimentation. Mice with catheters were individually housed in environmentally enriched cages with AL access to water and food. Catheters were flushed with sterile saline and refilled with sterile heparin glycerol locking solution (SAI Infusion Technologies, HGS) every 5–6 days.

Where indicated, axitinib was delivered via daily oral gavage at a dose of 25 mg/kg in 0.5% carboxymethylcellulose vehicle. To block VEGF/VEGFR2 signaling, mice were treated with DC101, a rat monoclonal IgG1 antibody against VEGFR2 (30 μg/kg, i.p., BioXcell) every other day for the duration of the dietary intervention.

To label proliferating cells, an i.p. injection of 5-ethynyl-2′-deoxyuridine (EdU) (E10187, Thermo Fisher Scientific) solution (5 mg/mL in saline, 10 μl/g BW injected) was given 7 h before sacrificing the mice.

### Method details

#### Exercise experiments

For endurance exercise capacity testing, mice were acclimated to a treadmill system (5-lane treadmill, Harvard Apparatus, Panlab) for 3 sessions on consecutive days before exercise capacity testing. All acclimation and testing were performed at 5% incline. During acclimation sessions each animal ran for 10 min, increasing the speed from 5 m/min up to 10 m/min by minute 5 and kept constant at 10 m/min from minute 5 to minute 10. Thereafter the animal rested for 5 min, followed by 10 min at 10 m/min. Following acclimation, mice underwent an aerobic exercise capacity test to exhaustion. Mice were motivated to run with a shock grid set at 0.2 mA. Starting speed was 5 m/min for 5 min and was increased by 1 m/min until exhaustion. Speed was capped at a maximum of 20 m/min until exhaustion. The electric current was turned off after a mouse received 10 electrical shocks. The treadmill test was terminated if the mice failed to return to the treadmill after 3 consecutive attempts within the last minute of running. Endurance exercise capacity is expressed as total time or distance run in meters during the test. Work performed during the one-time exercise test was calculated by multiplying the meters run by body weight as described previously.^68^ During metabolic treadmill experiments, the same protocol was followed but using Columbus Instruments metabolic treadmills to allow for measurement of gas exchange during the exercise testing.

#### Metabolic cages

Throughout the calorimetry studies, a standard 12-h light/dark cycle was maintained. Prior to data collection, all animals were weighed and acclimated to either control or SAAR diet for three days. Mice were placed in metabolic cages, and measurements began for seven consecutive days. Energy expenditure was determined using a computer-controlled indirect calorimetry system (PromethionH, Sable Systems, Las Vegas, NV) as published.^69^ Animals had unlimited access to food and water throughout the study. XYZ beam arrays (BXYZ-R, Sable Systems, Las Vegas, NV) were used to record ambulatory activity and position, and respiratory gasses were measured using an integrated fuel cell oxygen analyzer, a spectro-photometric CO_2_ analyzer, and a capacitive water vapor partial pressure analyzer (GA3, Sable Systems, Las Vegas, NV). Oxygen consumption and CO_2_ production were monitored for 1-min at 5-min intervals. The respiratory quotient (RQ) was determined by dividing CO_2_ production by O_2_ consumption. The Weir equation was used to calculate energy expenditure: Kcal/h = 60∗(0.003941∗VO_2_ + 0.001106∗VCO_2_). MetaScreen v. 2.5 was used to coordinate data acquisition and instrument control, and raw data was processed using ExpeData v. 1.8.5 (Sable Systems, Las Vegas, NV) via an analysis macro that detailed all aspects of data transformation.

#### Body composition and food intake

Body mass was determined by daily measurement at approximately ZT22. Lean and fat mass were measured in awake mice using an EchoMRI 100H body composition analyzer.

#### RNA extraction and quantitative RT-PCR

Tissues were harvested on day seven after dietary intervention (without running) and animals were fasted for 6 h prior to sacrifice. RNA sequencing was performed after 7 days of SAAR feeding in different muscle tissues, or after SAAR feeding and treatment with axitnib in EDL only. RNA of tissues was extracted using a RNeasy Kit according to the manufacturer’s instructions (QIAGEN, 74034). RNA purity and concentration were assessed via a spectrophotometer (Tecan, Spark or NanoDrop, ThermoFisher). RNA was reverse-transcribed to cDNA by High Capacity cDNA Reverse Transcription Kit (Thermo Fisher, 43-688-13). An SYBR Green-based master mix (ThermoFisher Scientific, A25778) was used for real-time qPCR analysis with primers listed in the key resources table. To compensate for variations in RNA input and efficiency of reverse-transcription, RPLP and Actin were used as a housekeeping gene. The delta-delta CT method was used to normalize the data.

#### RNA sequencing and differential gene expression analysis

RNA sequencing was performed by Novogene. The quality and quantity of isolated RNA and final libraries were determined using Qubit Fluorometer and Tapestation (Agilent, Waldbronn, Germany). Sequencing libraries were prepared following SMARTerÒ Universal Low Input RNA Kit for Sequencing. Briefly, total RNA samples (0.25–10 ng) were reverse-transcribed using random priming into double-stranded cDNA in the presence of a template switch oligo (TSO). Ribosomal cDNA was cleaved by ZapR in the presence of the mammalian-specific R-Probes. Remaining fragments were enriched with a second round of PCR amplification using primers designed to match Illumina adapters. The product is a smear with an average fragment size of approximately 360 bp. The libraries were normalized to 10 nM in Tris-Cl 10 mM, pH8.5 with 0.1% Tween 20. Read quality was assessed using FastQC. Alignment to the GRCm38 mouse reference genome was performed using the align function and annotation was performed using the featureCounts function from the Rsubread package.^70^ Genes were filtered based on minimum expression (>5 counts per million in at least 5 samples). Differential gene expression was computed using a negative binomial model implemented in the DESeq and limma packages.^71^^,^^72^ Significantly differentially expressed genes were defined as a *p*-value <0.01 with a false discovery ratio (FDR) < 0.1. FDR values were calculated using the Benjamini–Hochberg method. Gene Ontology Pathway analysis was performed using the clusterProfiler R package.^73^ Over-representation analysis was performed using the differentially expressed genes (DEGs). Geneset-enrichment was performed using 3 databases: GO Biological process (BP), KEGG pathway and Reactome pathway. *p*-values were corrected for multiple testing using the Benjamini-Hochberg procedure and adjusted *p*-values <0.05 were considered significant. Complex heatmaps were generated using the ComplexHeatmap package for R.^74^

#### Immunoblot analysis

Tissues were harvested on day seven after dietary intervention (without running) and animals were fasted for 6 h prior to sacrifice. Tissues were collected and lysed with [50 mM Tris–HCl pH 7.0, 270 mM sucrose, 5 mM EGTA, 1 mM EDTA, 1 mM sodium orthovanadate, 50 mM glycerophosphate, 5 mM sodium pyrophosphate, 50 mM sodium fluoride, 1 mM DTT, 0.1% Triton X-100 and a complete protease inhibitor tablet (Roche Applied Science)]. Lysates were centrifuged at 10000 g for 10 min at 4C. Supernatant was collected, and protein concentration was measured using the Pierce BCA protein assay kit (23225, ThermoFisher Scientific). 5–10 mg of total protein was loaded in a 15-well precast, gradient gel (456–8086, Bio-Rad). Proteins were transferred onto a PVDF membrane (Bio-rad, 170–4156) with a semi-dry or wet system and subsequently blocked for 1 h at room temperature with 5% milk in 0.1% TBS-Tween. Membranes were incubated overnight at 4C with primary antibodies listed in key resources table. The appropriate HRP-linked secondary antibodies (see key resources table) were used for chemiluminescent detection of proteins. Membranes were scanned with a Chemidoc imaging system (Bio-rad) and quantified using ImageJ software.

#### Immunohistochemistry and histology

Tissues were harvested on day seven after dietary intervention (without running) and animals were fasted for 6 h prior to sacrifice. EDL or soleus muscle samples were harvested and embedded in Tissue-Tek O.C.T. Compound, frozen in liquid N_2_-cooled isopentane and stored at −80 C until further use. Frozen muscle cross sections (7–10 μm) were made using a cryostat (Leica CM 1950) and collected on Superfrost Ultra Plus slides (Thermo Fisher Scientific). After acclimatizing to room temperature for approximately 15 min, skeletal muscle cryosections (10 μm) were fixed in 4% PFA for 10 min, washed three times with PBS and subsequently incubated for 1 h in blocking buffer (PBS with 10% donkey serum) at room temperature. Thereafter, samples were incubated overnight at 4C with primary antibodies diluted in blocking buffer with or without addition of 0.1% Triton X-100. Slides were subsequently washed in PBS and incubated for 1 h in blocking buffer with the appropriate secondary antibodies at 1:250 dilution. Nuclei were stained with Hoechst.

Images were captured with a Zeiss Axio observer Z.1 or an Olympus confocal microscope (FV1200). Fiber cross-sectional area was automatically determined on laminin-stained sections with the Muscle J plugin for ImageJ software.^75^ In the axitinib experiment, vascular density (% CD31^+^ area) was quantified within the whole tissue with ImageJ software after threshold processing on 20x images acquired with a Nikon Eclipse Ti2 microscope.

#### Stable isotope infusions

For intravenous infusions, U-^13^C_18_-linoleate (Cambridge Isotope Laboratories) was prepared at a 2 mM concentration in saline with 1 mM BSA. Mice underwent surgery to insert a jugular vein catheter and were allowed to recover for at least one week before experiments. The infusion setup (Instech Laboratories) included a swivel and tether to allow the mouse to move around the cage freely. Infusion rate was set to 0.3 μL/min and tracer infused for 90 min followed by tail blood collection and tissue harvesting. Fasted infusions were collected at 5PM 8 h after chow removal (the infusion was started at 2:30PM).

#### Serum metabolite extraction

Serum (3 μL) was extracted with cold 100% methanol (40-fold dilution), vortexed, and incubated on dry ice for 30 min. Then, the extract was centrifuged at 20,000 x g for 20 min at 4°C and supernatant was transferred to new tubes and diluted 5-fold with 100% methanol, vortexed and incubated on dry ice for 30 min. Then, the extract was centrifuged at 20,000 x g for 20 min at 4°C and supernatant was transferred to tubes for LC-MS analysis.

#### Tissue metabolite extraction

Frozen tissue pieces were pulverized using a Cryomill (Retsch) at cryogenic temperature. Ground tissue was weighed (10–20 mg) and transferred into a precooled tube for extraction. Soluble metabolites extraction was done by adding cold 40:40:20 methanol:acetonitrile:water to the tissue powder (40 μL solvent per mg tissue). Samples were vortexed for 10 s, incubated on wet ice for 20 min and then centrifuged at 4 °C at 20,000 x g for 30 min. Supernatant was transferred to LC-MS vials for analysis.

#### Metabolite measurement by LC-MS

LC−MS analysis for soluble metabolites was achieved on a quadrupole-orbitrap mass spectrometer (Thermo Scientific): the Q Exactive PLUS hybrid, Exploris 240 or Exploris 480. Each mass spectrometer was coupled to hydrophilic interaction chromatography (HILIC) via electrospray ionization. To perform the LC separation of serum and tissue samples, an XBridge BEH Amide column (150 mm × 2.1 mm, 2.5 μM particle size, Waters) was used with a gradient of solvent A (95%:5% H2O: acetonitrile with 20 mM ammonium acetate, 20 mM ammonium hydroxide, pH 9.4), and solvent B (100% acetonitrile). The gradient was 0 min, 85% B; 2 min, 85% B; 3 min, 80% B; 5 min, 80% B; 6 min, 75% B; 7 min, 75% B; 8 min, 70% B; 9 min, 70% B; 10 min, 50% B; 12 min, 50% B; 13 min, 25% B; 16 min, 25% B; 18 min, 0% B; 23 min, 0% B; 24 min, 85% B; 30 min, 85% B. The flow rate was 150 μL min−1, an injection volume of 10 μL for serum samples and 5 μL for tissue samples, and column temperature was 25°C. MS full scans were in negative or positive ion mode with a resolution of 140,000 at m/z 200 and scan range of 70–1,000 m/z. The automatic gain control (AGC) target was 1 × 106. LC-MS peak files were analyzed and visualized with El-MAVEN (Elucidata) using 5 ppm ion extraction window, minimum peak intensity of 1 x 105 ions, and minimum signal to background blank ratio of 2. For infusion experiments, the software package Accucor was used to correct for metabolite labeling from natural isotope abundance.

#### Circulatory flux measurements

Circulatory flux (Fcirc) was calculated from isotope enrichment at 60 min of tracer infusion, following previously published protocols that have established pseudo–steady state conditions under similar settings.^43^ While our study did not include serial measurements to experimentally confirm steady state, the consistency with prior work supports the assumption that steady state was approximated under our experimental conditions. The fraction of the fully labeled tracer (i.e., the infused form), L_M+C_ (for example, linoleate is M+18 due to having 18 carbon atoms) was used:

(Equation 1) Fcirc=R·L[M+C]L[M+C]

where R is the infusion rate of the labeled tracer. Since the circulatory flux is a pseudo-steady state measurement, for minimally tracer infusions, production flux is approximately equal to consumption flux of the metabolite and thus *F*_circ_ reflects both the circulating production and consumption fluxes of the infused metabolite.

#### Tissue lipid extraction

Frozen tissue pieces were pulverized using a Cryomill (Retsch) at cryogenic temperature. Soluble lipid extraction was done by adding 50% methanol:50% H_2_O and chloroform 2:1 to the samples. Samples were incubated on wet ice for 10min and then centrifuged at 4°C at 20,000 x *g* for 5 min. The bottom layer was extracted using glass hamilton syringes and transferred to glass vials for further processing. The first extraction step was repeated, and the chloroform was evaporated using a nitrogen gas manifold. Samples were reconstituted in 1:1:1 methanol, acetonitrile, isopropyl alcohol for analysis.

#### Lipid measurement by LC-MS

Lipids were analyzed using a Vanquish Horizon UHPLC System (Thermo Scientific) coupled to a Q Exactive Plus mass spectrometer (Thermo Scientific). Agilent Poroshell 120 EC-C18 column (particle size, 2.7 μm; 150 mm (length) × 2.1 mm (i.d.)) was used for separation. Column temperature was 25 °C. Mobile phases A = 1 mM ammonium acetate and 0.2% (v/v) acetic acid in 90:10 (v/v) water:methanol and B = 1 mM ammonium acetate and 0.2% (v/v) acetic acid in 98:2 (v/v) isopropanol:methanol were used for ESI positive mode. The linear gradient eluted from 25% B (0.0–2.0 min), 25% B to 65% B (2.0–4.0 min), 65% B to 100% B (4.0–16.0 min), 100% B (16.0–20.0 min), 100% B to 25% B (20.0–21.0 min), 25% (21.0–25.0 min). The flow rate was 0.15 mL/min. The sample injection volume was 5 μL. ESI source parameters were as follows: spray voltage, 3200 V or −2800 V, in positive or negative modes, respectively (arb = arbitrary units); sheath gas, 35 arb; aux gas, 10 arb; sweep gas, 0.5 arb; ion transfer tube temperature, 300 °C; vaporizer temperature, 35 °C. LC-MS data acquisition was operated under full scan positive mode for all samples. The full scan was set as: orbitrap resolution, 70,000 at m/z 200; AGC target, 3e6 arb; maximum injection time, 250 ms; scan range, 265–1150 m/z. LC-MS peak files were analyzed and visualized with El-MAVEN (Elucidata) using 5 ppm ion extraction window, minimum peak intensity of 1 x 105 ions, and minimum signal to background blank ratio of two. For infusion experiments, the software package Accucor was used to correct for metabolite labeling from natural isotope abundance.

#### *Ex vivo* beta oxidation assay

Tissues were harvested on day seven after dietary intervention (without running) and animals were fasted for 6 h prior to sacrifice. After seven-day dietary treatment mice fed either a Con or SAAR diet were sacrificed and EDL and soleus dissected, weighed and immediately put on ice in low glucose DMEM (Thermo Fisher Scientific). To start the assay, muscles are transferred to low glucose DMEM media containing 2% fatty acid free BSA, 0.25 mM carnitine and 2 μCi/ml [9,10-3H]-palmitic acid (NET53100, PerkinElmer, Zaventem, Belgium). Tissues were incubated for 3 h in culture medium at 37 C and 5% CO_2_, after which the supernatant was taken, and 10% Trichloroacetic acid (TCA) added and incubated at room temperature for 15 min. Samples were spun down at max speed for 10 min before 5% TCA was added followed by 10% BSA in TE buffer. After 15 min of incubation samples were spun down again and the supernatant was incubated with Chloroform:Methanol (2:1) and KCl:HCl was added. Samples were spun down on last time, before the supernatant was transferred into scintillation vials and 3H labeling was determined using a b-counter. CPM values were background subtracted and normalized to mg wet weight of the tissue. During beta-oxidation, ^3^H-palmitate undergoes enzymatic cleavage, releasing ^3^H-labeled water (^3^H-H_2_O) as a by-product. Following incubation, the medium was collected, and ^3^H-H_2_O was separated and quantified using liquid scintillation counting. The amount of ^3^H-H_2_O released reflects the degree of fatty acid oxidation, providing a measure of mitochondrial oxidative capacity.

#### Isolation of endothelial cells (ECs)

Primary ECs from skeletal muscle (mECs) were isolated from adult WT and EC^CD36-/-^ littermates. Mice were euthanized, all hindlimb muscles were immediately dissected, and muscles were minced in a Petri dish on ice using a surgical blade. Next, the minced muscle tissue was enzymatically digested in digestion buffer containing 2 mg/mL Dispase II (D4693, Sigma-Aldrich), 2 mg/mL Collagenase IV (17104019, Thermo Fisher Scientific) and 2 mM CaCl_2_ in PBS at 37°C for 40 min, with gentle shaking every 10 min. The reaction was stopped by adding an equal volume of 20% FBS in HBSS and the suspension was passed through a series of 100-μm cell strainers (Corning) and 70-μm cell strainers (Corning) to remove tissue debris. After a series of centrifugation and washing steps, the heterogeneous cell population was purified by FACS.

#### Flow cytometry

Cells were incubated in PBS with the fixable viability dye eFluor 780 (65-0865-14, eBioscience) before antibody staining. Prior to surface staining with antibodies, Fc gamma receptors were blocked by incubating cells with anti-CD16/CD32 antibodies (2.4G2, homemade). Thereafter, cells were incubated with the appropriate primary antibodies (CD45, CD31, CD36) diluted in FACS buffer (DPBS +2% FCS) and subsequently incubated with antibodies for 30 min on ice. For EdU proliferation experiments, cells from EdU-injected mice were first stained with antibodies for cell surface markers and subsequently labeled with the click-iT plus EdU Alexa Fluor 488 Flow Cytometry Assay Kit (Life Technologies) according to the manufacturer’s instructions. Cells were analyzed with a LSRFortessa (BD Bioscience) flow cytometer or sorted using a FACS Aria III (BD Bioscience) sorter. Data were analyzed using FlowJo 10 software (Tree Star). A complete list of all antibodies and staining reagents used can be found in key resources table. The gating strategies used for flow cytometry plots are shown in Figure S3.

#### Enzyme-linked immunosorbent assay (ELISA)

Tissues were harvested on day seven after dietary intervention (without running) and animals were fasted for 6 h prior to sacrifice. Skeletal muscle tissue samples (10–15 mg) were homogenized with a tissue homogenizer (Omni THq) in ice-cold lysis buffer (1:15 w/v) as described above. Homogenates were centrifuged at 10000 g for 10 min at 4C, and VEGF was measured in the supernatants using the Mouse VEGF Quantikine ELISA Kit (R&D System, MMV00) according to the manufacturer’s protocol.

### Quantification and statistical analysis

The images presented in the manuscript are representative of the data (quantification of image is approximately the group average) and the image/staining quality. All data represent mean ± standard deviation (SD). GraphPhad Prism software (version 8.0.0) was used for statistical analyses. Investigators were always blinded to group allocation. When comparing two group means, Student’s *t* test was used in an unpaired two-tailed fashion. For more than two groups, one-way ANOVA with Tukey’s multiple comparisons test was used and for experimental set-ups with a second variable, two-way ANOVA with Sidak’s multiple comparisons test was used. The statistical method used for each experiment is indicated in each figure legend. No experiment-wide multiple test correction was applied. *p* > 0.05 is considered non-significant. *p* < 0.05 is considered significant.

## Supplemental information

Document S1. Figures S1–S5 and Tables S1

Table S2. Raw data from metabolic treadmill measurements Data supplied in an excel file as it is too large for a PDF or word table.

Table S3. Raw counts data from RNA sequencing analysis looking at muscle and diet interaction Data supplied in an excel file as it is too large for a PDF or word table.

Table S4. Raw normalized ion counts from lipidomics analysis looking at muscle and diet interaction Data supplied in an excel file as it is too large for a PDF or word table.

Table S5. Raw normalized ion counts from metabolomics analysis looking at muscle and diet interaction Data supplied in an excel file as it is too large for a PDF or word table.

Table S6. Raw normalized ion counts from metabolomics analysis looking at muscle and diet interaction in different tissues from WT and CD36^EC−/−^ mice Data supplied in an excel file as it is too large for a PDF or word table.

Table S7. Raw counts data from RNA sequencing analysis looking at diet and axitinib treatment interaction in EDL muscle Data supplied in an excel file as it is too large for a PDF or word table.

Data S1. Raw images for Figure 2E western blot scans
