# Supplementary material for: Angiogenesis-independent VEGF signaling enhances exercise capacity by increasing fat oxidation in mice fed sulfur amino acid-restricted diets
Source: iScience. 2025 Nov 20;28(12):114148. doi: 10.1016/j.isci.2025.114148 (PMC12721204; doi:10.1016/j.isci.2025.114148)
Supplement: Document S1. Figures S1–S5 and Tables S1 [file mmc1.pdf]

**iScience, Volume *28***

## Supplemental information

# Angiogenesis-independent VEGF signaling enhances exercise capacity by increasing fat oxidation in mice fed sulfur amino acid-restricted diets

**Charlotte G. Mann, Michael R. MacArthur, Jing Zhang, Songlin Gong, Jenna E. AbuSalim, Craig J. Hunter, Wenyun Lu, Thomas Agius, Alban Longchamp, Florent Allagnat, Joshua D. Rabinowitz, James R. Mitchell, Katrien De Bock, and Sarah J. Mitchell**

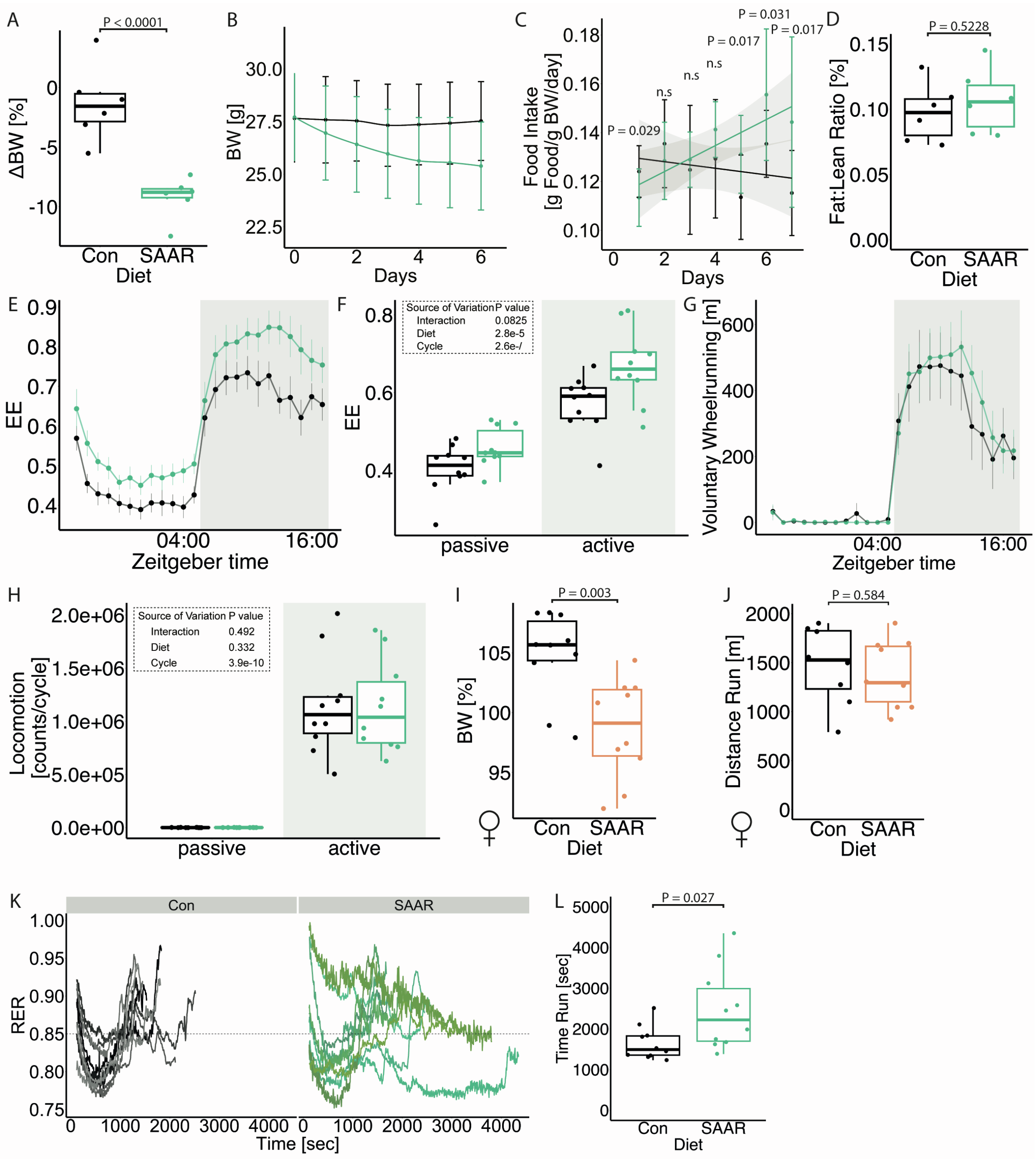
A
P < 0.0001
ΔBW [%]
Con
SAAR
Diet
B
BW [g]
Days
C
Food Intake
[g Food/g BW/day]
P = 0.029
n.s
n.s
n.s
P = 0.017
P = 0.031
P = 0.017
Days
D
P = 0.5228
Fat:Lean Ratio [%]
Con
SAAR
Diet
E
EE
Zeitgeber time
04:00
16:00
F
Source of Variation
P value
Interaction
0.0825
Diet
2.8e-5
Cycle
2.6e-7
EE
passive
active
G
Voluntary Wheelrunning [m]
Zeitgeber time
04:00
16:00
H
Source of Variation
P value
Interaction
0.492
Diet
0.332
Cycle
3.9e-10
Locomotion
[counts/cycle]
passive
active
I
P = 0.003
BW [%]
Con
SAAR
Diet
J
P = 0.584
Distance Run [m]
Con
SAAR
Diet
K
Con
SAAR
RER
Time [sec]
L
P = 0.027
Time Run [sec]
Con
SAAR
Diet

**Supplemental Figure 1. Short-term SAAR induces shifts in metabolism and increases endurance exercise capacity in young, sedentary male mice. Related to main Figure 1 and Supplemental Table 2.** (**A**) Change in percent body weight when compared to starting body weight (n = 6) of male mice given *ad libitum* access to sulfur amino acid restricted (SAAR) versus control (Con) diet for seven days. Percent fat mass (**B**), percent lean mass (**C**) of total body weight measured using ECHO MRI and fat:lean mass ratio (**D**) (n = 6) of male mice given *ad libitum* access to SAAR versus Con diet on day seven. (**E**) Change in percent body weight when compared to starting body weight (n = 8) of female mice given *ad libitum* access to SAAR versus Con diet after seven days on the diet. (**F**) Distance ran during a one-time maximal endurance test (n = 8) of female mice given *ad libitum* access to SAAR versus Con diet. Sable systems indirect calorimetry measurements of energy expenditure (**G**) (kcal, EE) over a 24 h period and the average EE during a 12 h–12 h light–dark cycle (**H**) (n = 10) of male mice given *ad libitum* access to SAAR versus Con diet on day seven. Sable systems indirect calorimetry measurements of sum of beam breaks (**I**) (counts/cycle, Locomotion) and of voluntary wheel running behavior (**J**) over a 24 h period (n = 10) of male mice given *ad libitum* access to SAAR versus Con diet for seven days. (**K**) Linear regression showing the relationship between relative change in body weight versus distance ran and (**L**) Linear regression showing the relationship between absolute body weight versus distance ran (n = 16) of male mice given *ad libitum* access to SAAR versus Con diet on day seven. $R^2$ coefficient was calculated using Pearson's method. (**M**) RER trajectory over time in seconds during a one-time maximal endurance test on metabolic treadmills (Harvard Apparatus) and (**N**) time ran in seconds during a one-time maximal endurance test measured on metabolic treadmills (n = 10) of male mice given *ad libitum* access to SAAR versus Con diet on day seven. Panels A-D,G-J represent data from mice that were not subjected to endurance running, panels E-F,K-N represent data from mice subjected to maximal endurance testing. All data is shown as mean and error bars indicate SD unless otherwise noted; p values indicate the significance of the difference by Student's t test or two-way ANOVA with Sidak's multiple comparisons test between diets or diet and cycle (indirect calorimetry); significance is determined by a p value of $p < 0.05$. For linear regressions r squared Pearson's coefficient was calculated.

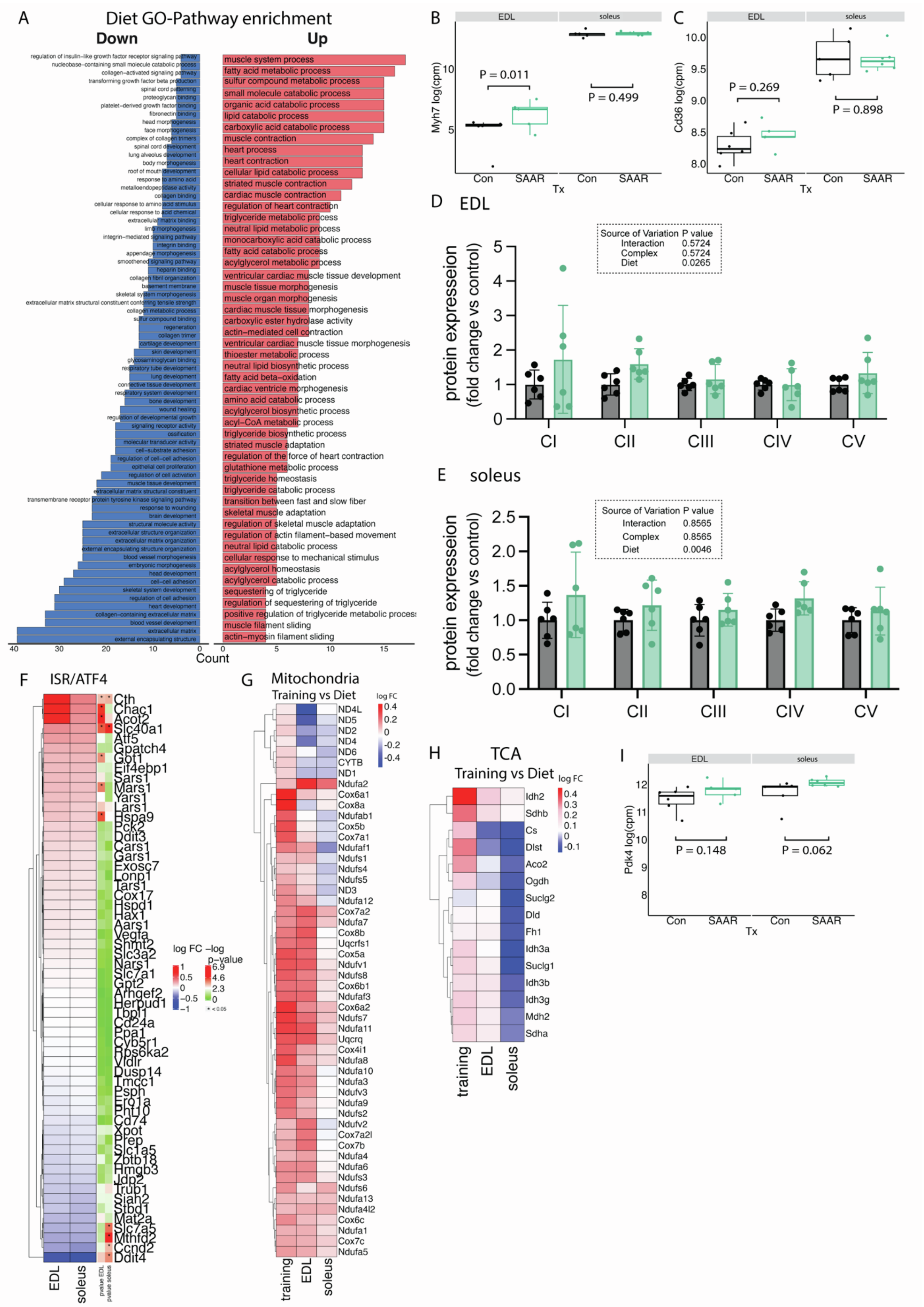

A
Diet GO-Pathway enrichment
Down
Up
Count
B
C
D EDL
E soleus
F ISR/ATF4
G Mitochondria
Training vs Diet
H TCA
Training vs Diet
I

**Supplemental Figure 2. Transcriptomics across muscle depots reveal metabolic shift from glycolytic toward oxidative. Related to main Figure 2 and Supplemental Table 3.** (**A**) Pathway enrichment analysis encompassing transcript datasets comparing main dietary effects after bulk RNA sequencing (n = 6) of male mice given *ad libitum* access to sulfur amino acid restricted (SAAR) versus control (Con) diet on day seven showing all significantly increased or decreased pathways. Log count values of the transcript Myh7 (**B**) and Cd36 (**C**) in both EDL and soleus after bulk RNA sequencing (n = 6) of male mice given *ad libitum* access to SAAR versus Con diet on day seven. Quantification of relative protein abundance normalized to vinculin of all five complexes of the electron transport chain from blots shown in Figure 2E of both EDL (**D**) and soleus (**E**) (n = 5). (**F**) Fold changes of transcripts associated with known dietary SAAR and integrated stress response (ISR) target genes [S1] after SAAR when compared to Con. Fold changes after Training [S2] and SAAR when compared to Con of specific genes associated with mitochondrial matrix genes (**G**) or genes associated with TCA cycle as identified Figure 2E (**H**). All panels represent data from mice that were not subjected to endurance running. All data is shown as mean and error bars indicate SD unless otherwise noted; p values indicate the significance of the difference by Student's t test or two-way ANOVA with Sidak's multiple comparisons test between diets or diet and complexes; significance is determined by a p value of $p < 0.05$.

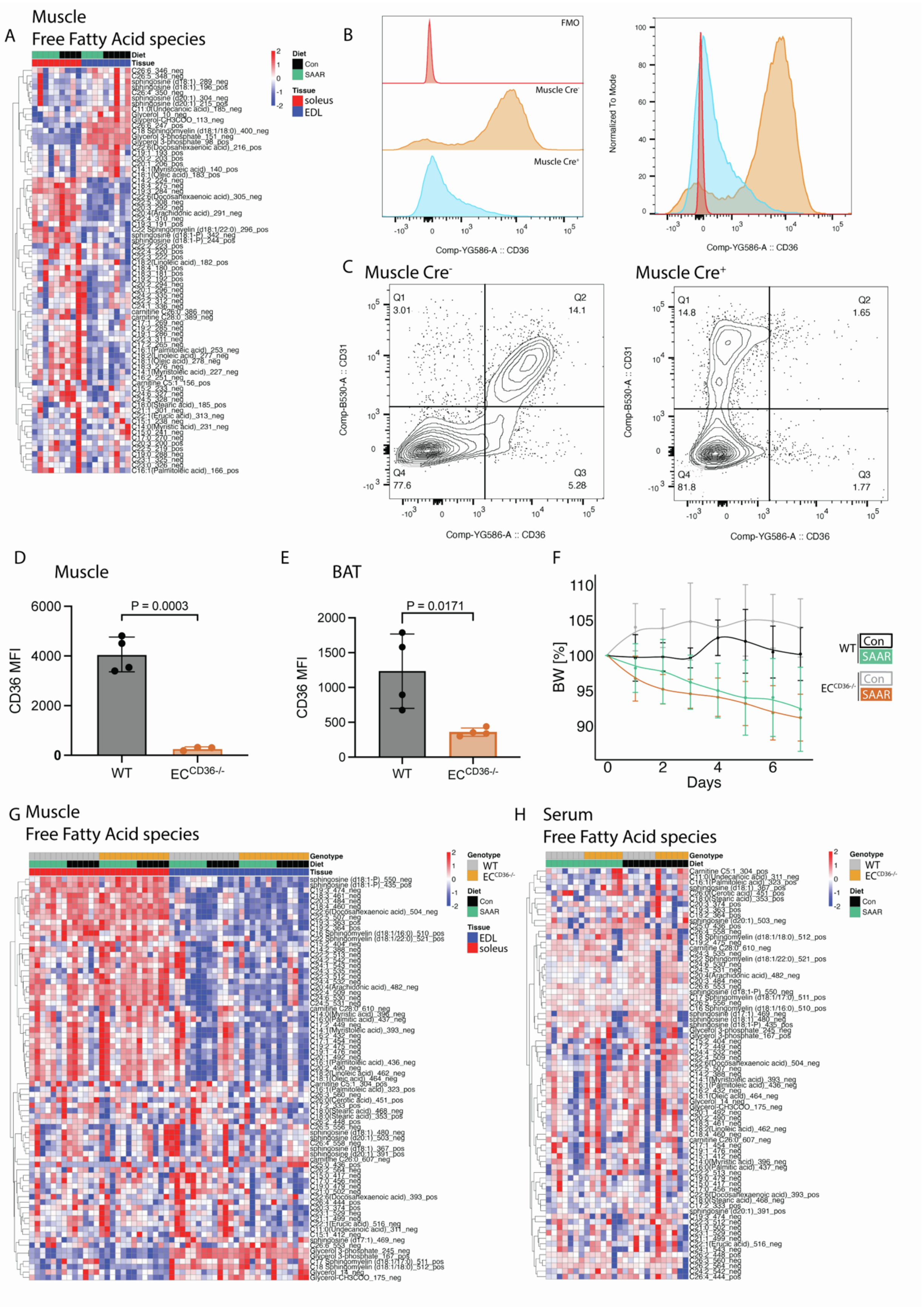

A
Muscle
Free Fatty Acid species
Diet
Con
SAAR
Tissue
soleus
EDL
B
FMO
Muscle Cre⁻
Muscle Cre⁺
Normalized To Mode
Comp-YG586-A :: CD36
C
Muscle Cre⁻
Muscle Cre⁺
Q1
3.01
Q2
14.1
Q3
5.28
Q4
77.6
Q1
14.8
Q2
1.65
Q3
1.77
Q4
81.8
Comp-B530-A :: CD31
Comp-YG586-A :: CD36
D
Muscle
P = 0.0003
CD36 MFI
WT
EC CD36-/-
E
BAT
P = 0.0171
CD36 MFI
WT
EC CD36-/-
F
BW [%]
Days
WT
Con
SAAR
EC CD36-/-
Con
SAAR
G
Muscle
Free Fatty Acid species
Genotype
Diet
Tissue
WT
EC CD36-/-
Con
SAAR
EDL
soleus
H
Serum
Free Fatty Acid species
Genotype
Diet
WT
EC CD36-/-
Con
SAAR

**Supplemental Figure 3. SAAR increases muscle lipid flux without altering lipid pool sizes. Related to main Figure 3 and Supplemental Tables 4-6.** (**A**) Heatmap of differentially abundant free fatty acid species in muscles (n= 3-4) of male mice given *ad libitum* access to sulfur amino acid restricted (SAAR) versus control (Con) diet for seven days. Representative histograms of $CD36^{+}$ endothelial cells (EC) in the muscle (**B**) and gating strategy (**C**) for CD36 positive EC ($CD45^{-}$, $CD31^{+}$, $CD36^{+}$) isolated from muscle of male WT ($Cre^{-}$) and $EC^{CD36-/-}$ ($Cre^{+}$) mice. $EC^{CD36-/-}$ KO efficiency was confirmed by FACS analysis of $CD31^{+}$/$CD36^{+}$ MFI in muscle (**D**) or brown adipose tissue (BAT) (**E**) (n = 4) of male WT or $EC^{CD36-/-}$ mice. (**F**) Daily body weight trajectories shown in percent of starting body weight (n = 8/group) over time of male WT and $EC^{CD36-/-}$ mice given *ad libitum* access to SAAR versus Con diet for seven days. Heatmap of differentially abundant free fatty acid species in muscles (**G**) or serum (**H**) of male WT or $EC^{CD36-/-}$ mice (n = 5) fed a Con or SAAR diet for seven days. All panels represent data from mice that were not subjected to endurance running. All data is shown as mean and error bars indicate SD unless otherwise noted; p values indicate the significance of the difference by Student's t test between diets, or two-way ANOVA with Sidak's multiple comparisons test between diets and muscle or genotype; significance is determined by a p value of $p < 0.05$.

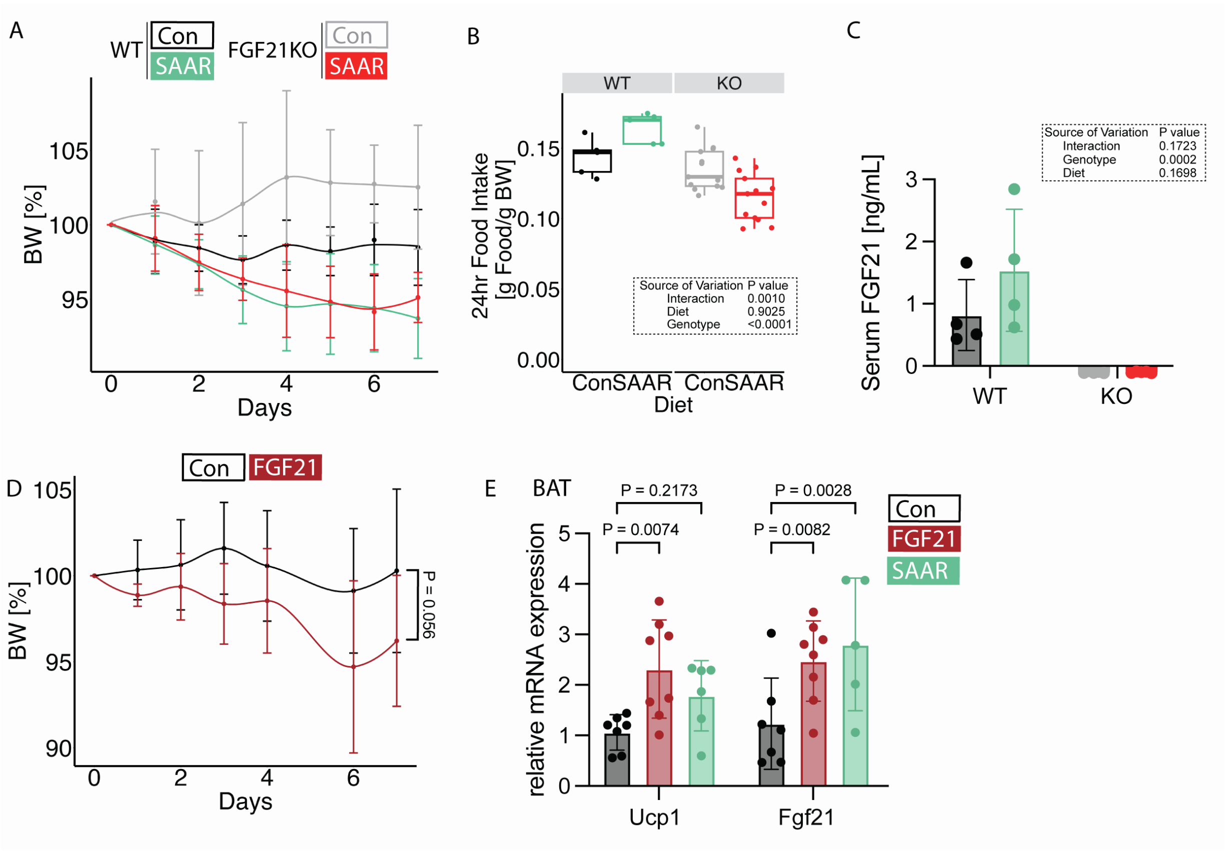


**Supplemental Figure 4. FGF21 is dispensable for running phenotype after SAAR in male mice. Related to main Figure 4.** (**A**) Daily body weight trajectories shown in percent of starting body weight (n = 4 - 10) over time, of male WT or FGF21KO mice given *ad libitum* access to sulfur amino acid restricted (SAAR) versus control (Con) diet for seven days. (**B**) Food intake expressed as grams of food eaten per gram of mouse body weight within a 24 hr period (n = 4 - 10) of male WT or FGF21KO mice given *ad libitum* access to SAAR versus Con diet for seven days. (**C**) Serum FGF21 concentrations of male WT or FGF21 KO mice given *ad libitum* access to SAAR versus Con diet for seven days determined using an ELISA. (**D**) Daily body weight trajectories over time shown in percent when compared to starting body weight (n = 8) of NaCl or recombinant FGF21 treated male mice for seven days. (**E**) Fgf21 and Ucp1 mRNA levels in brown adipose tissue (BAT) of male mice given *ad libitum* access to SAAR versus Con diet or mice treated with recombinant FGF21 for seven days. Panels A-C represent data from mice that were not subjected to endurance running, panels D-E represent data from mice subjected to maximal endurance testing. All data is shown as mean and error bars indicate SD unless otherwise noted; p values indicate the significance of the difference by Student's t test between diets, or two-way ANOVA with Sidak's multiple comparisons test between diets and genotype; significance is determined by a p value of $p < 0.05$.

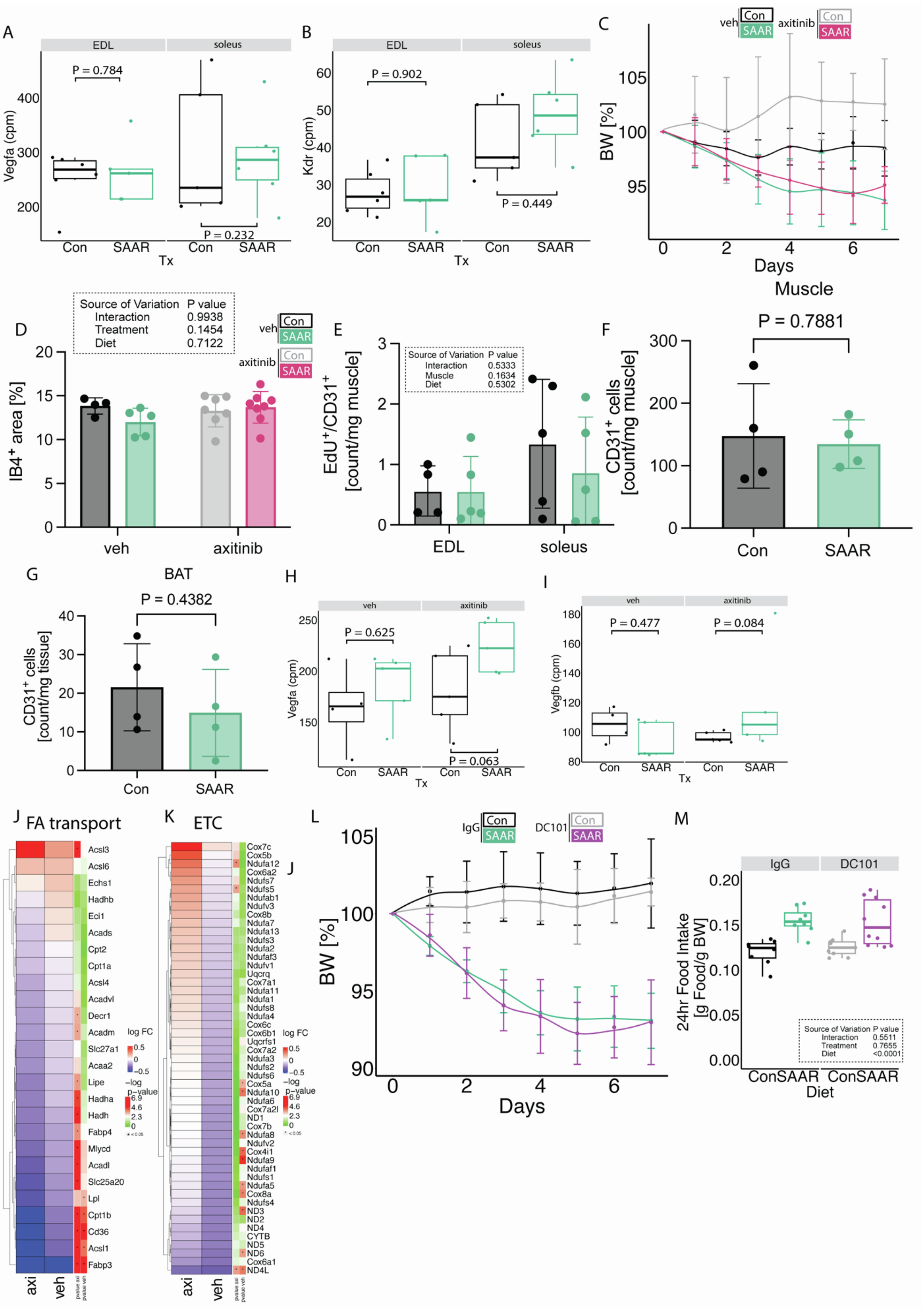
A
EDL
soleus
P = 0.784
P = 0.232
Vegfa (cpm)
Con
SAAR
Tx
B
P = 0.902
P = 0.449
Kdr (cpm)
C
veh
axitinib
Con
SAAR
BW [%]
Days
D
Source of Variation P value
Interaction 0.9938
Treatment 0.1454
Diet 0.7122
IB4+ area [%]
veh
axitinib
E
Source of Variation P value
Interaction 0.5333
Muscle 0.1634
Diet 0.5302
EdU+/CD31+ [count/mg muscle]
EDL
soleus
F
Muscle
P = 0.7881
CD31+ cells [count/mg muscle]
G
BAT
P = 0.4382
CD31+ cells [count/mg tissue]
H
P = 0.625
P = 0.063
Vegfa (cpm)
I
P = 0.477
P = 0.084
Vegfb (cpm)
J FA transport
Acsl3
Acsl6
Echs1
Hadhb
Eci1
Acads
Cpt2
Cpt1a
Acsl4
Acadvl
Decr1
Acadm
Slc27a1
Acaa2
Lipe
Hadha
Hadh
Fabp4
Mlycd
Acadl
Slc25a20
Lpl
Cpt1b
Cd36
Acsl1
Fabp3
log FC
−log p−value
axi
veh
K ETC
Cox7c
Cox5b
Ndufa12
Cox6a2
Ndufs7
Ndufs5
Ndufab1
Ndufv3
Cox8b
Ndufa7
Ndufa13
Ndufs3
Ndufa2
Ndufaf3
Ndufv1
Uqcrq
Cox7a1
Ndufa11
Ndufa1
Ndufs8
Ndufa4
Cox6c
Cox6b1
Uqcrfs1
Cox7a2
Ndufa3
Ndufs2
Ndufs6
Cox5a
Ndufa10
Ndufa6
Cox7a2l
ND1
Cox7b
Ndufa8
Ndufv2
Cox4i1
Ndufa9
Ndufaf1
Ndufs1
Ndufa5
Cox8a
Ndufs4
ND3
ND2
ND4
CYTB
ND5
ND6
Cox6a1
ND4L
L
IgG
DC101
Con
SAAR
BW [%]
Days
M
IgG
DC101
24hr Food Intake [g Food/g BW]
Source of Variation P value
Interaction 0.5511
Treatment 0.7655
Diet <0.0001
ConSAAR ConSAAR
Diet

**Supplemental Figure 5. Inhibition of VEGFR signaling prevents endurance exercise phenotype without induction of angiogenesis. Related to main Figure 4 and Supplemental Table 7.** Count values of the transcript Vegfa (**A**) and Kdr (**B**) in both EDL and soleus after bulk RNA sequencing (n = 6) of male mice given *ad libitum* access to sulfur amino acid restricted (SAAR) versus control (Con) diet on day seven using transcriptomic dataset presented in figure 2. (**C**) Daily body weight trajectories shown in percent of starting body weight (n = 10) over time of male mice given *ad libitum* access to SAAR versus Con diet for seven days, treated with either vehicle (veh) or axtinib by oral gavage. (**D**) Quantification of $IB4^+$ area of EDL muscle of male mice fed a Con or SAAR for seven days treated with veh or axitinib (n = 5-8). (**E**) Cell counts of $EdU^+/CD31^+$ double positive cells per mg tissue in muscle from male mice given *ad libitum* access to SAAR versus Con diet for seven days and injected with EdU to label cell proliferation, determined by flow cytometry. Cell counts of $CD31^+$ positive cells per mg tissue in muscle (**F**) or brown adipose tissue (BAT) (**G**) or of male mice given *ad libitum* access to SAAR versus Con diet for seven days, determined by flow cytometry. Count values of the transcript Vegfa (**H**) or Kdr (**I**) in both EDL treated with veh or axitinib after bulk RNA sequencing (n = 5) of male mice given *ad libitum* access to SAAR versus Con diet on day seven. (**J**) Fold changes of transcripts associated with fatty acid (FA) catabolism and transport as identified in supplementary figure 2A in both EDL treated with veh or axitinib after bulk RNA sequencing (n = 5) of male mice given *ad libitum* access to SAAR versus Con diet on day seven. (**K**) Fold changes of transcripts associated with fatty acid (FA) catabolism and transport as identified in supplementary figure 2A in both EDL treated with veh or axitinib after bulk RNA sequencing (n = 5) of male mice given *ad libitum* access to SAAR versus Con diet on day seven. (**L**) Body weight trajectory over time, shown as percent of starting body weight (n = 8-10) of male mice given *ad libitum* access to SAAR versus Con diet for seven days treated with IgG or DC101 via i.p. injection every other day. (**M**) Food intake expressed as grams of food per gram of body weight per mouse within a 24 hr period (n = 8-10) of male mice given *ad libitum* access to SAAR versus Con diet treated with IgG or DC101 via i.p. injection every other day on day seven. Panels A-K represent data from mice that were not subjected to endurance running, panels L-M represent data from mice subjected to maximal endurance testing. All data is shown as mean and error bars indicate SD unless otherwise noted; p values indicate the significance of the difference by Student’s t test between diets, or two-way ANOVA with Sidak’s multiple comparisons test between diets and muscle or treatment; significance is determined by a p value of $p < 0.05$.

**Supplemental Table 1. Amino Acid composition of Research Diet used throughout the study.**

| **Product #** | A17101101 | | A17101103 | |
|---|---|---|---|---|
| | Control | | Low Methionine | |
| | **% gram** | **% kCal** | **% gram** | **% kCal** |
| Protein | 16.8 | 18 | 16.4 | 17 |
| Carbohydrate | 68.8 | 72 | 69.2 | 73 |
| Fat | 4.3 | 10 | 4.3 | 10 |
| Total | 89.9 | 100 | 89.9 | 100 |
| **kCal/gram** | 3.8 | | 3.8 | |
| **Ingredient** | **gram** | | **gram** | |
| Casein, Lactic | 0 | | 0 | |
| L-Cystine | 0 | | 0 | |
| L-Isoleucine | 7.6 | | 7.6 | |
| L-Leucine | 15.8 | | 15.8 | |
| L-Lysine | 13.2 | | 13.2 | |
| L-Methionine | 4.5 | | 1.2 | |
| L-Phenylalanine | 8.4 | | 8.4 | |
| L-Threonine | 7.2 | | 7.2 | |
| L-Tryptophan | 2.1 | | 2.1 | |
| L-Valine | 9.3 | | 9.3 | |
| L-Histidine-HCl-H2O | 4.6 | | 4.6 | |
| L-Alanine | 5.1 | | 5.1 | |
| L-Arginine | 6 | | 6 | |
| L-Aspartic Acid | 12.1 | | 12.1 | |
| L-Glutamic Acid | 38.2 | | 38.2 | |
| Glycine | 3 | | 3 | |
| L-Proline | 17.8 | | 17.8 | |
| L-Serine | 10 | | 10 | |
| L-Tyrosine | 9.2 | | 9.2 | |
| Total L-Amino Acids | 174.1 | | 170.8 | |
| Corn Starch | 506.2 | | 506.2 | |
| Maltodextrin 10 | 125 | | 125 | |
| Sucrose | 73.6 | | 73.6 | |
| Cellulose, BW200 | 50 | | 50 | |
| Soybean Oil | 25 | | 25 | |
| Lard | 20 | | 20 | |
| Mineral Mix S10026 | 10 | | 10 | |
| DiCalcium Phosphate | 13 | | 13 | |
| Calcium Carbonate | 5.5 | | 5.5 | |
| Potassium Citrate, 1 H2O | 16.5 | | 16.5 | |
| Vitamin Mix V10001 | 10 | | 10 | |
| Choline Bitartrate | 2 | | 2 | |

| | | |
|---|---|---|
| Sodium Bicarbonate | 7.5 | 7.5 |
| FD&C Yellow Dye #5 | 0.05 | 0 |
| FD&C Red Dye #40 | 0 | 0.05 |
| **Total** | **1038.45** | **1038.45** |

## Supplemental References


[S1] Torrence ME, MacArthur MR, Hosios AM, Valvezan AJ, Asara JM, Mitchell JR, Manning BD. The mTORC1-mediated activation of ATF4 promotes protein and glutathione synthesis downstream of growth signals. Davis RJ, Ron D, Shen K, eds. *eLife*. 2021;10:e63326. doi:10.7554/eLife.63326

[S2] Furrer R, Heim B, Schmid S, Dilbaz S, Adak V, Nordström KJV, Ritz D, Steurer SA, Walter J, Handschin C. Molecular control of endurance training adaptation in male mouse skeletal muscle. *Nat. Metab*. 2023;5:2020-2035. doi:10.1038/s42255-023-00891-y
