# Supplementary material for: Angiogenesis-independent VEGF signaling enhances exercise capacity by increasing fat oxidation in mice fed sulfur amino acid-restricted diets
Source: iScience. 2025 Nov 20;28(12):114148. doi: 10.1016/j.isci.2025.114148 (PMC12721204; doi:10.1016/j.isci.2025.114148)
Supplement: Data S1. Raw images for Figure 2E western blot scans [file mmc8.zip › Data S1/Data S1 .docx]

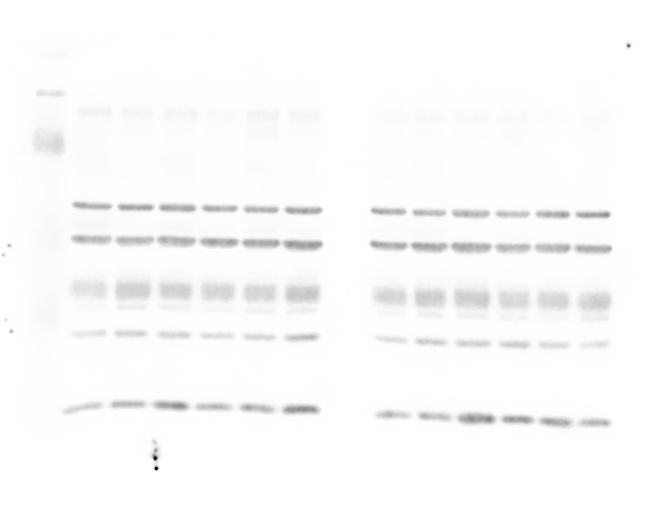

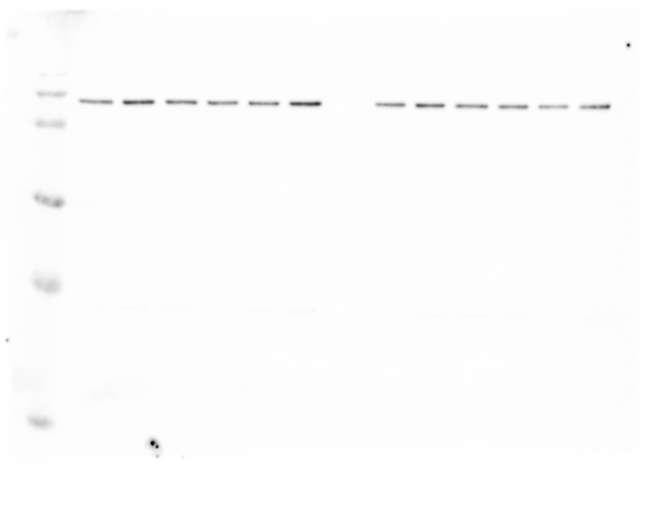

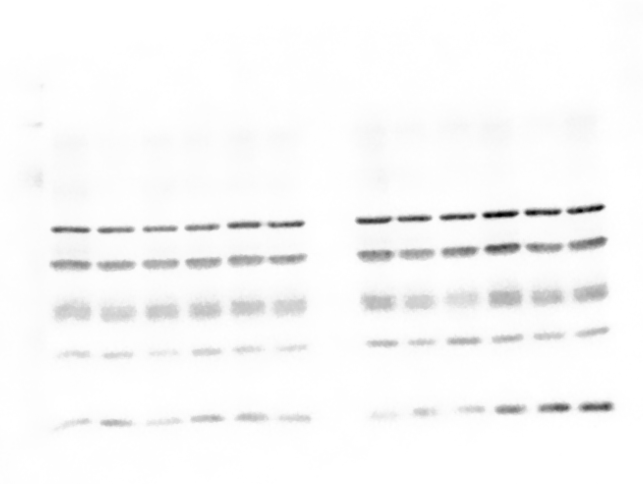

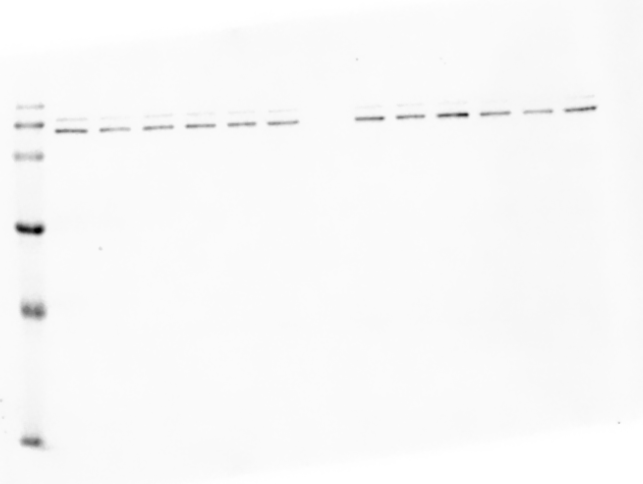


Figure 4 raw file of anti OXPHOS staining of Soleus con vs SAAR (correlates to Fig2E BOTTOM

Figure 3 raw file of anti-vinculin staining of Soleus con vs SAAR (correlates to Fig2E BOTTOM)

Figure 1 raw file of anti-vinculin staining of EDL con vs SAAR (correlates to Fig2E TOP)

Figure 2 raw file of anti OXPHOS staining of EDL con vs SAAR (correlates to Fig2E TOP
