# Supplementary figures and images for: Angiogenesis-independent VEGF signaling enhances exercise capacity by increasing fat oxidation in mice fed sulfur amino acid-restricted diets
Source: iScience. 2025 Nov 20;28(12):114148. doi: 10.1016/j.isci.2025.114148 (PMC12721204; doi:10.1016/j.isci.2025.114148)

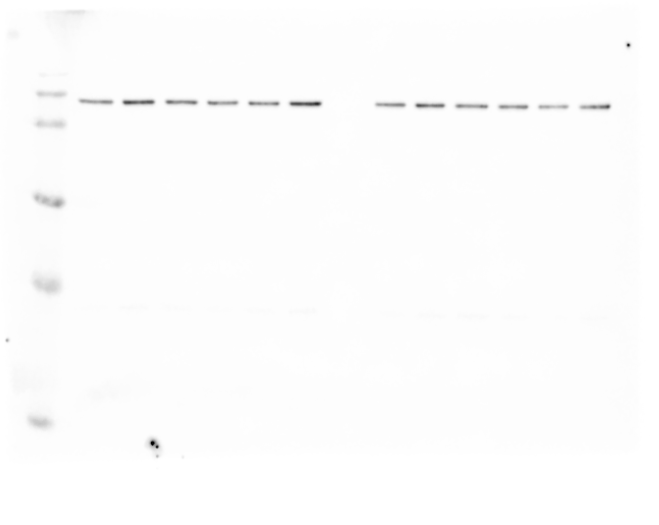

Supplement: Data S1. Raw images for Figure 2E western blot scans [file mmc8.zip › Data S1/sol oxphos 191021 vincullin.tif]

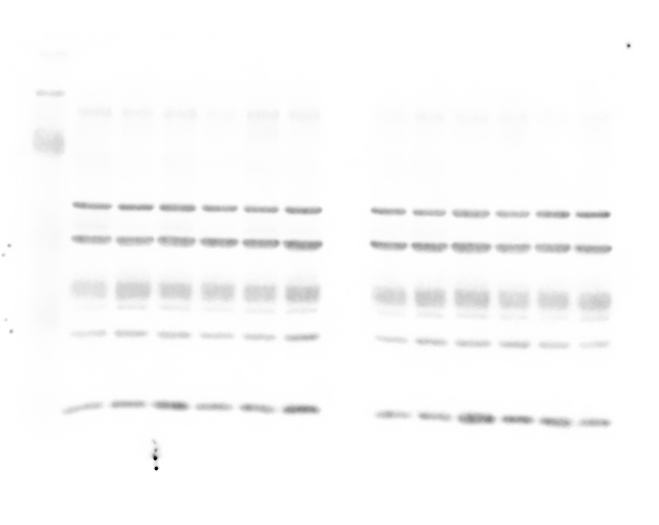

Supplement: Data S1. Raw images for Figure 2E western blot scans [file mmc8.zip › Data S1/sol oxphos 191021 oxphos.tif]

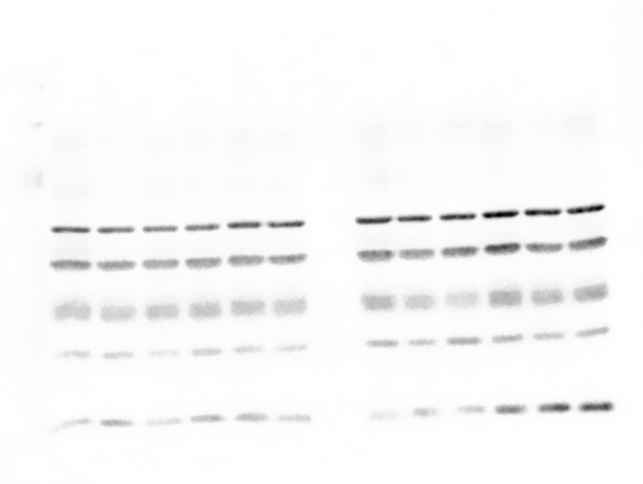

Supplement: Data S1. Raw images for Figure 2E western blot scans [file mmc8.zip › Data S1/edl oxphos 191021 oxphos.tif]

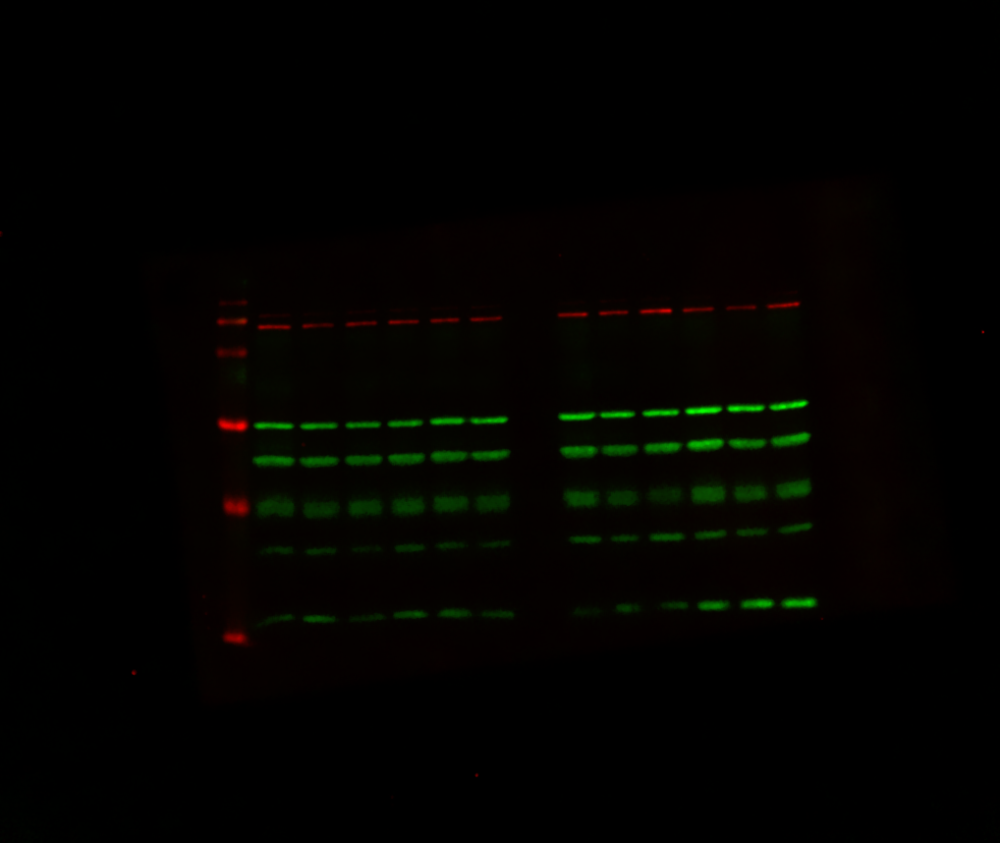

Supplement: Data S1. Raw images for Figure 2E western blot scans [file mmc8.zip › Data S1/edl oxphos 191021.tif]

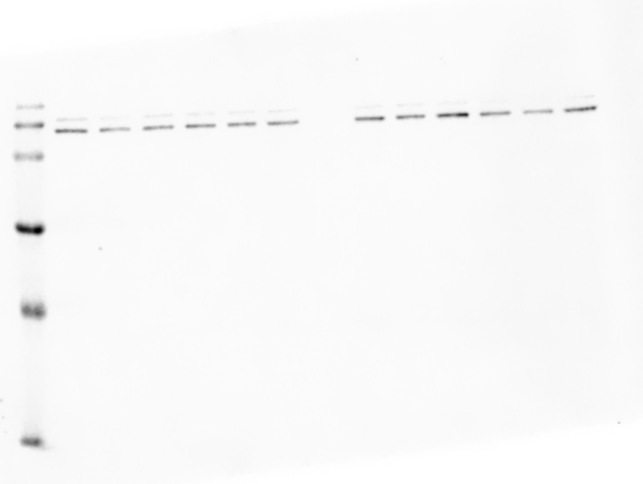

Supplement: Data S1. Raw images for Figure 2E western blot scans [file mmc8.zip › Data S1/edl oxphos 191021 vincullin.tif]

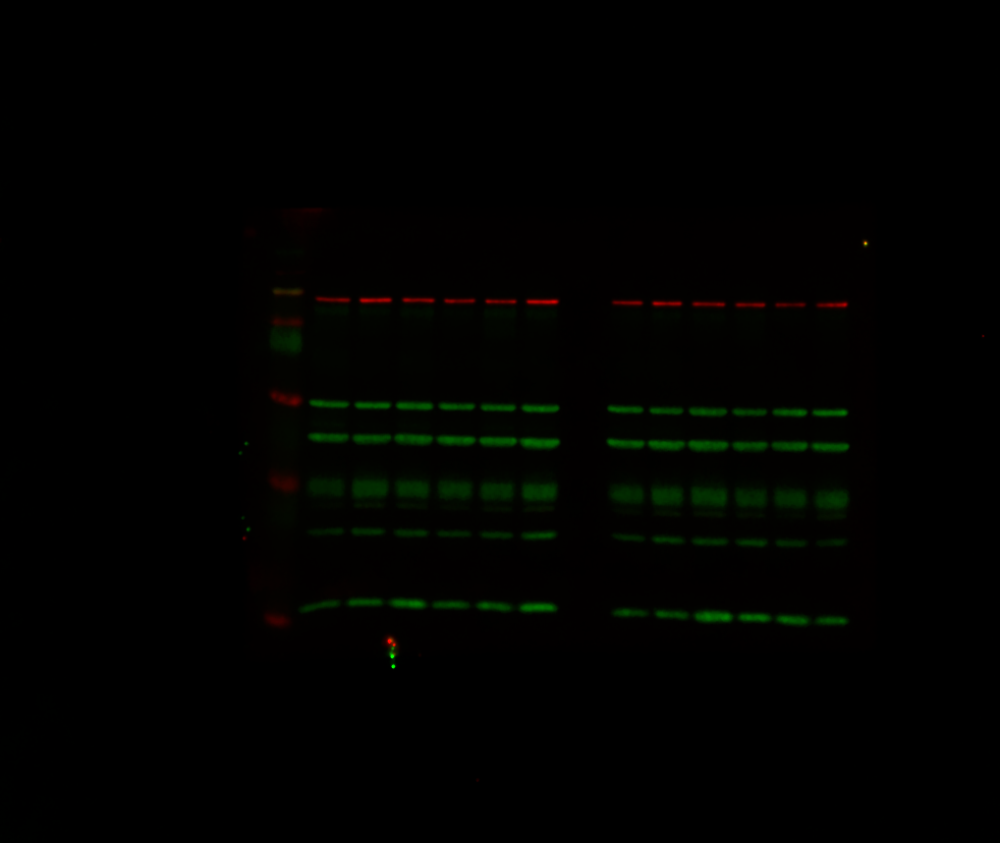

Supplement: Data S1. Raw images for Figure 2E western blot scans [file mmc8.zip › Data S1/sol oxphos 191021.tif]
